# ﻿Re-evaluation of *Ceratostomella* and *Xylomelasma* with introduction of two new species (Sordariomycetes)

**DOI:** 10.3897/mycokeys.110.136844

**Published:** 2024-11-21

**Authors:** Martina Réblová, Jana Nekvindová, Miroslav Kolařík, Željko Jurjević, Michal Kolář, Vít Hubka

**Affiliations:** 1 Department of Taxonomy, Institute of Botany, The Czech Academy of Sciences, 252 43 Průhonice, Czech Republic Institute of Botany, The Czech Academy of Sciences Průhonice Czech Republic; 2 Institute of Clinical Biochemistry and Diagnostics, University Hospital Hradec Králové, 500 05 Hradec Králové, Czech Republic University Hospital Hradec Králové Hradec Králové Czech Republic; 3 Laboratory of Fungal Genetics and Metabolism, Institute of Microbiology, The Czech Academy of Sciences, 142 20 Prague 4, Czech Republic Institute of Microbiology, The Czech Academy of Sciences Prague Czech Republic; 4 EMSL Analytical, Inc., 200 Route 130 North, Cinnaminson, New Jersey 08077, USA EMSL Analytical, Inc. Cinnaminson United States of America; 5 Laboratory of Genomics and Bioinformatics, Institute of Molecular Genetics, The Czech Academy of Sciences, 142 20 Prague 4, Czech Republic Institute of Molecular Genetics, The Czech Academy of Sciences Prague Czech Republic; 6 Department of Botany, Faculty of Science, Charles University, 128 00 Prague 2, Czech Republic Charles University Prague Czech Republic

**Keywords:** Ascogenous hyphae, biogeography, cryptic species, molecular systematics, saprobes, Sordariomycetes, two new species

## Abstract

In this study, we assessed the phylogenetic relationships among members of *Ceratostomella* and the morphologically similar genus *Xylomelasma*, currently classified within the Sordariomycetes. Our phylogenetic analyses, utilising three and five gene markers, revealed that species from these two genera are congeneric, supporting the transfer of *Xylomelasma* to *Ceratostomella*. Consequently, we propose two new combinations: *C.sordida***comb. nov.** and *C.novae-zelandiae***comb. nov.** In addition, we identified two cryptic species within the *C.sordida* species complex, which are described as *C.crypta***sp. nov.** and *C.melanospora***sp. nov.** Traditional micromorphological characters have proven insufficient for differentiating these new species; however, they are clearly distinguishable by molecular data, particularly using the internal transcribed spacer region ITS1-5.8S-ITS2 (ITS) of the nuclear rRNA cistron, and genes encoding the second largest subunit of RNA polymerase II (*rpb2*), and translation elongation factor 1-α (*tef1-α*) as primary and secondary barcodes. This study provides new insights into the morphological characteristics of *Ceratostomella*, identifying the ascogenous system as an important diagnostic trait at the generic level, which distinguishes *Ceratostomella* from morphologically similar fungi. *Ceratostomella* is currently recognised with eight species. We also investigated the relationship between *Ceratostomella* and the closely related *Barbatosphaeria*. The lack of statistical support in the Maximum likelihood analysis is discussed and the inclusion of *Ceratostomella* in Barbatosphaeriaceae is not supported. *Ceratostomella* is accepted as a genus *incertae sedis*, while Barbatosphaeriaceae remains a monotypic family. The global diversity of *Ceratostomella* is inferred from metabarcoding data and published field observations. Biogeographic analysis indicates that members of *Ceratostomella* are widespread, found in soil and decaying wood, as well as in air, dust, roots, shoots, and water across temperate, subtropical and tropical regions in both the Northern and Southern Hemispheres. We are concurrently publishing whole-genome analyses of three ex-type strains of *Ceratostomella*, i.e. *C.crypta*, *C.melanospora* and *C.sordida*. This effort aims to establish a new standard for high-quality taxonomic studies, which, in accordance with current trends, should incorporate whole-genome sequencing data for future research and application. Our findings underscore the importance of integrating morphological, biogeographic and molecular data for accurate species delineation and highlight the complexity within the genus *Ceratostomella*.

## ﻿Introduction

*Ceratostomella* (Saccardo 1878) was introduced to encompass species with simple diagnostic features, such as dark rostrate ascomata and hyaline, aseptate ascospores. According to MycoBank (Crous et al. 2004), 111 species have historically been assigned to this genus. However, the following studies have revealed that *Ceratostomella* is phenotypically more diverse than previously thought (e.g. Sydow and Sydow 1919; Elliott 1923; Melin and Nannfeldt 1934; Moreau 1952; Hunt 1956; Booth 1957; Dennis 1988; Samuels and Barr 1997; Réblová 2006; De Beer et al. 2013; Réblová et al. 2018). It includes taxa with both evanescent and persistent asci, as well as hyaline or pigmented ascospores. Additionally, it encompasses various types of interthecial filaments and, where known, asexual morphs characterised by annellidic, holoblastic, phialidic, and tretic conidiogenesis. The enormous species variability within *Ceratostomella* has posed a challenging task for mycologists, who have made numerous attempts to classify its species. Various studies using molecular data (see below) have demonstrated that *Ceratostomella* represents a heterogeneous species assemblage exhibiting phenotypic convergence. Despite their similar phenotypes, these species belong to several phylogenetically distinct lineages.

Of the numerous species ascribed to *Ceratostomella*, only a handful represents the core of the genus. Réblová (2006) redefined *Ceratostomella* through comparative morphology and phylogenetic analyses of ribosomal DNA sequences from two representative species, *C.cuspidata* and *C.pyrenaica*. Molecular data for the lectotype species, *C.rostrata*, designated by Clements and Shear (1931), are not available. *Ceratostomella* is characterised by non-stromatic perithecial ascomata with an upright cylindrical, sulcate neck, thick leathery to carbonaceous ascomatal wall, persistent clavate asci arising from supportive, discrete ascogenous cells, usually inconspicuous apical annulus, broadly cellular paraphyses, and brown, aseptate, suballantoidal, irregularly ellipsoidal, globose or reniform ascospores arranged in fascicle or 2–3-seriately within the ascus. The asexual morphs are unknown, and the axenic cultures derived from ascospores remained sterile. *Ceratostomella* thrives in strongly decayed wood or decaying *Polyporales* basidiomata and is primarily distributed in temperate regions of both hemispheres. The genus was classified as *incertae sedis* within *Sordariomycetidae* with four species accepted (Réblová 2006), and later, together with *Barbatosphaeria* and *Xylomelasma*, placed in the family Barbatosphaeriaceae by Zhang et al. (2017).

The revision of other *Ceratostomella* species with persistent asci prompted their reclassification in several unrelated genera within Sordariomycetes. These include *Barbatosphaeria* in Barbatosphaeriaceae (Réblová 2008; Réblová et al. 2015b), *Calyptosphaeria*, *Lentomitella*, *Spadicoides* and *Torrentispora* in *Xenospadicoidales* (Réblová et al. 2018), *Ceratosphaeria* in *Magnaporthales* (von Niessl 1876), *Chaetosphaeria* in Chaetosphaeriales (Booth 1957; Huhndorf and Fernández 2005), *Clohiesia* in *Annulatascales* (Réblová et al. 2018), *Daruvedia* in *Pyrenulales* (Dennis 1988), *Natantiella* (*incertae sedis*) (Réblová and Štěpánek 2009), *Jattaea* and *Togniniella* in Calosphaeriales (Réblová et al. 2004, 2015a; Réblová 2011), *Phaeoacremonium* in Togniniales (Gramaje et al. 2015), *Phomatospora* in *Phomatosporales* (Réblová et al. 2018), *Pseudorhynchia* in *Hypocreales* (Samuels and Barr 1997), and *Wallrothiella* in *Amplistromatales* (Réblová and Seifert 2004). Other species with evanescent asci and hyaline ascospores have been reclassified into several genera such as *Ceratocystis*, *Huntiella* and *Thielaviopsis* of *Microascales* and *Ceratocystiopsis*, *Grosmannia*, *Leptographium*, *Ophiostoma* and *Pesotum* of *Ophiostomatales*; these species including their synonymy are listed in De Beer et al. (2013, 2014). Unravelling the phylogenetic relationships of many other *Ceratostomella* species remains a complex task. This complexity arises from the lack of living cultures, insufficient diagnoses, ambiguous or lost holotypes, and the scarcity of recent collections. The entire taxonomic history of *Ceratostomella* was detailed in Réblová et al. (2018).

*Xylomelasma* (Réblová 2006) is remarkably similar to *Ceratostomella* in both morphology and ecology. The genus was typified by *X.sordida*, and *X.novae-zelandiae* was accepted as a second species. Both genera share similar ascospores and persistent asci that float freely within the centrum at maturity, robust paraphyses, rostrate non-stromatic ascomata with thick wall and occasional occurrence of Munk pores in the wall cells. In addition, they exhibit identical ascogenous apparatus, consisting of oval, clavate or obclavate, discrete cells from which asci arise as an outgrowth. *Xylomelasma* was distinguished from *Ceratostomella* by its ellipsoidal ascospores that are obliquely uniseriate, occasionally biseriate in the ascus, more or less cylindrical paraphyses, distinct apical annulus and ascomata with both sulcate and non-sulcate necks. Based on single-gene nuclear large and small subunit rDNA (LSU and SSU) phylograms, *Ceratostomella* and *Xylomelasma* were shown to be closely related, although they did not form a monophyletic clade (Réblová 2006). However, in the phylogenetic analyses based on the concatenated data set of LSU, SSU, and the second largest subunit of RNA polymerase II (*rpb2*) DNA sequences, a strongly supported relationship between the two genera was confirmed (Réblová et al. 2018). Currently, *Xylomelasma* contains four species, including *X.moderata* (Vassiljeva and Stephenson 2014) and *X.shoalensis* (Hernández-Restrepo et al. 2016).

During a survey of ascomycetes, we collected specimens of *C.cuspidata* and *C.pyrenaica* from Belgium and New Zealand, along with four collections preliminarily identified as *X.sordida* from wood in advanced stages of decay in the Czech Republic, and from a swab from generating station in the USA. Axenic cultures were derived from all collections. Furthermore, we re-examined the holotype of *X.novae-zelandiae*, which lacks a living culture as the ascospores never germinated *in vitro*, and we successfully extracted DNA from the ascomata.

The aim of this study is to investigate the relationships between *Ceratostomella* and *Xylomelasma*, as well as among the four ‘*X.sordida*’ strains isolated by us, using comparative morphological studies and novel DNA sequences. Additionally, we sought to re-evaluate the morphological traits originally used to delimit both genera. To achieve this, we generated new sequences for nuclear rDNA ITS1-5.8S-ITS2 (ITS barcode), nuclear LSU and SSU rDNA, *rpb2*, and the intermediate section of the coding region of translation elongation factor 1-α (*tef1-α*) from all available ex-type and non-type strains of *Ceratostomella* and *Xylomelasma* and subjected them to phylogenetic analyses.

In our integrative taxonomic approach, we combine both phylogenetic and morphological data. With the capability of utilising GlobalFungi (Větrovský et al. 2020), we also incorporate geographical and ecological information. A crucial next step in advancing taxonomic standards and refining fungal classification is the concurrent publication of whole-genome sequence (WGS) data for type strains. This approach allows for more comprehensive comparisons between taxa, including with those so-called ‘fungal dark taxa’—fungi that lack observable morphological structures, cannot be cultured under laboratory conditions, and are primarily detected through DNA sequencing, particularly via environmental metabarcoding (Nilsson et al. 2019). The current topic of significant discussion is the potential introduction of DNA-based typification (e.g. Nilsson et al. 2023), with WGS data serving as an alternative typification material. For such a system to function effectively, WGS data must also be available for the currently described diversity. In response to the call by Zhou (2024), we publish short-read WGS data for representative strains of *Ceratostomella*, supporting this emerging trend in fungal taxonomy.

## ﻿Materials and methods

### ﻿Fungal strains and morphological studies

Material was obtained from temperate regions in both the Northern and Southern Hemispheres, including a swab from the USA and collections on wood from temperate broadleaf and mixed forests in Belgium, Czech Republic, and New Zealand. Dried specimens were deposited into the Fungarium of the Institute of Botany CAS (**PRA**) in Průhonice, Czech Republic and New Zealand Fungarium (**PDD**) in Auckland, New Zealand. Cultures were deposited in Westerdijk Fungal Biodiversity Institute (**CBS**) in Utrecht, the Netherlands, and Culture Collection of Fungi (**CCF**), Faculty of Science, Charles University in Prague, Czech Republic. Other herbarium material was obtained from Fungarium of the Illinois Natural History Survey (**ILLS**) in Champaign, Illinois, USA. Along with our collections and literature references, data on the host and geographic distribution of the species studied were obtained from the MyCoPortal (http://www.mycoportal.org/portal/index.php, Miller and Bates 2017, accessed on October 14, 2024). Taxonomic novelties were registered in MycoBank. Table 1 presents the studied strains, their sources, and the GenBank accession numbers for the sequences obtained in this study.

**Table 1.** Taxa, isolate information and new sequences determined for this study (in bold) and additional sequences retrieved from GenBank.

Taxon	Source	Status	Country	Host	Substrate	GenBank accession numbers					Ref.
						ITS	nucLSU	nucSSU	*rpb2*	*tef1-α*	
*Ceratostomellacrypta*	CBS 131683	T	Czech Republic	unidentified	decayed wood	KT991679	KM492871	KM492860	KM492910	**PQ213498**	1,2
*C.crypta*	CBS 131684	P	Czech Republic	unidentified	decayed wood	**PQ215754**	**PQ215747**	**PQ215922**	**PQ213489**	**PQ213499**	
*C.crypta*	CCF 5710	P	USA	n/a	generating station (swab)	**PQ215755**	**PQ215748**	**PQ215923**	—	—	
*Ceratostomellacuspidata*	ICMP 17629		New Zealand	*Nothofagus* sp.	decayed wood/bark	KT991671	FJ617558	KT991642	KT991651	**PQ213500**	1,3
*C.cuspidata*	IFBL 57.31		Belgium	unidentified	decayed wood	**PQ215756**	**PQ215749**	**PQ215924**	**PQ213490**	**PQ213501**	
*Ceratostomellamelanospora*	CBS 147993	T	Czech Republic	*Fagussylvatica*	decayed wood	**PQ215757**	**PQ215750**	**PQ215925**	**PQ213491**	**PQ213502**	
*Ceratostomellanovae-zelandiae*	PDD 81433	T	New Zealand	*Nothofagus* sp.	decayed wood/bark	**PQ215758**	**PQ215751**	**PQ215926**	—	—	
*Ceratostomellapyrenaica*	CBS 117116	P	Czech Republic	*Acercampestre*	decayed wood	**PQ215759**	DQ076323	DQ076324	**PQ213492**	**PQ213503**	4
*C.pyrenaica*	PRA-21825		Czech Republic	*Quercus* sp.	decayed wood	**PQ215760**	**PQ215752**	**PQ215927**	—	**PQ213504**	
*C.pyrenaica*	CBS 129343		Czech Republic	*Quercus* sp.	decayed wood	KT991672	KY931835	KY931893	KY931863	—	2,4
*Ceratostomellasordida*	CBS 116000	T	France	*Alnusglutinosa*	decayed wood	**PQ215761**	AY761087	AY761090	KY931929	**PQ213505**	4,5
*Neotracyllapini*	CBS 146010	T	Malaysia	*Pinustecunumanii*	needles	—	**PQ215753**	**PQ215928**	**PQ213493**	—	
*Tracyllaaristata*	CBS 141404	E	Australia	*Eucalyptusregnans*	leaves	—	OL654186	**PQ215929**	**PQ213494**	—	6
*Tracyllaeucalypti*	CBS 144429	T	Colombia	*Eucalyptusurophylla*	leaves	—	OL654187	**PQ215930**	**PQ213495**	—	6

Notes: T, E, P indicate ex-holotype, ex-epitype and ex-paratype strains. References: 1 = Réblová et al. 2015b; 2 = Réblová et al. 2016; 3 = Réblová and Štěpánek 2009; 4 = Réblová et al. 2018; 5 = Réblová 2006; 6 = Réblová et al. 2021.

Structures of the fungi on the host and living cultures were examined with an Olympus SZX12 dissecting microscope (Olympus America, Inc., Melville, NY, USA). Dry ascomata were rehydrated with water, and the gelatinous centrum was extracted using the tip of a needle. Microscopic preparations were mounted in 90% lactic acid, water, and Melzer’s reagent. Measurements were taken from specimens mounted in Melzer’s reagent and means ± standard deviation (SD) were calculated for sizes of asci and ascospores based on a minimum of 20–25 measurements. Microscopic observations were conducted using an Olympus BX51 light microscope. Nomarski differential interference contrast (DIC) and phase contrast (PC) were used for observations and measurements. Microphotographs were captured using an Olympus DP70 camera with Imaging Software Cell^D (Olympus). Colony macrophotographs were captured with a Canon EOS 77D digital camera with Canon EF 100 mm f/2.8L Macro IS USM objective (Canon Europe Ltd., Middlesex, UK) with daylight spectrum 5500K 16W LED lights. Images were processed using Adobe Photoshop CS6 software (Adobe Systems, San Jose, CA, USA).

One isolate from a swab and five cultures derived from ascospores of fresh specimens were prepared in the context of this study; unfortunately, those of *C.cuspidata* IFBL 57.31 and *C.pyrenaica*PRA-21825 are no longer viable. Axenic cultures were prepared as outlined by Jurjević et al. (2015) and Réblová and Nekvindová (2023). In order to assess colony characteristics, diffusible pigments and growth patterns, strains were cultivated on cornmeal dextrose agar (CMD) (cornmeal agar, Oxoid Limited, Basingstoke, UK, supplemented with 2% w/v dextrose), MLA (Modified Leonian’s agar) (Malloch 1981), oatmeal agar (OA), and potato-carrot agar (PCA) (Crous et al. 2019a). In addition, other nutrient media such as Modified cellulose agar (MCA), malt extract agar (MEA), and potato-dextrose agar (PDA) (HealthLink, Jacksonville FL, USA; currently Hardy diagnostics), were also used to incubate cultures. To measure the size of the colonies *in vitro*, an agar plug with 2-week-old mycelium was placed at the centre and at the edge of new 9 cm and 6 cm Petri dishes. Colony diameter was measured from cultures that were two and four weeks old. Colony characteristics were determined based on 4-week-old cultures incubated in the dark at 23 °C. To assess the growth at higher temperatures, the cultures were incubated at 30, 35, 37 and 41 °C on MEA, PDA, and OA for a period of two weeks. To induce sporulation, strains were also inoculated on cornmeal agar (CMA, Crous et al. 2019a) supplemented with sterile stems of *Urticadioica* and exposed to alternating UV light and darkness in 12-hour intervals.

### ﻿Gene markers, DNA extraction, PCR amplification, and sequencing

The relationships between *Ceratostomella* and *Xylomelasma*, as well as intraspecific and interspecific relationships within *Ceratostomella* were evaluated using five gene markers. These include the internal transcribed spacer ITS1-5.8S-ITS2 of the nuclear rRNA cistron used as a primary barcode for fungi (Schoch et al. 2012); the nuclear large subunit rDNA gene (D1−D3 domains, approximately 1800 base pairs) and the nuclear small subunit rDNA gene, which are commonly employed for studying relationships within the *Ascomycota* at the generic and higher taxonomic levels (e.g. Schoch et al. 2009). Additionally, genes encoding the second largest subunit of RNA polymerase II (DNA-directed RNA polymerase) and the intermediate section of the translation elongation factor 1-α were used, as they are effective in distinguishing interspecific relationships in fungi (e.g. Robert et al. 2011; Stielow et al. 2015).

Protocols for DNA extraction and PCR amplification of ITS, LSU, SSU, *rpb2*, and *tef1-α* were conducted following the methods described by Réblová and Nekvindová (2023). Automated sequencing was carried out by Eurofins Genomics Europe Sequencing Service (Cologne, Germany). Analyses of raw sequence data and assembly of sequence contigs were performed using Sequencher v. 5.4.6 (Gene Codes Corp., Ann Arbor, MI, USA). In Suppl. material 1, we provide the accession numbers of sequences for members of Sordariomycetes obtained from GenBank, most of which have been previously published in other studies (Ranghoo et al. 1999; Suh and Blackwell 1999; Huhndorf et al. 2004; Réblová and Seifert 2004; Réblová et al. 2004, 2011, 2015a,b, 2016, 2018, 2020, 2021; Miller and Huhndorf 2005; Réblová 2006, 2011, 2013; Shenoy et al. 2006, 2010; Yaguchi et al. 2006; Zhang et al. 2006, 2017; Arzanlou et al. 2007; Spatafora et al. 2007; Damm et al. 2008; Plattner et al. 2009; Réblová and Štěpánek 2009; Thongkantha et al. 2009; Ferrer et al. 2012; Jaklitsch et al. 2013; Untereiner et al. 2013; Klaubauf et al. 2014; Tsang et al. 2014; Hernández-Restrepo et al. 2016; Senanayake et al. 2016; Yang et al. 2017; Réblová and Štěpánek 2018; Song et al. 2018; Crous et al 2019b; Luo et al. 2019; Lu et al. 2020; Hyde et al. 2021).

The ex-type strains of *C.crypta*CBS 131683, *C.melanospora*CBS 147993 and *C.sordida*CBS 116000 were selected for whole-genome DNA sequencing. Genomic DNA was extracted from 5-day-old cultures grown on MEA agar plates using the NucleoSpin® Soil isolation kit (Macherey–Nagel, Düren, Germany). Library preparation (2 × 300 bp Illumina paired-end) was carried out, and sequencing was performed on a NextSeq 2000 instrument (Illumina) following the manufacturer’s protocol. The quality of the raw sequencing data was assessed using FastQC v. 0.11.9 (Andrews 2010) (Accessed on 23 Aug. 2024), and low-quality reads were filtered out using Trimmomatic v. 0.39 (Bolger et al. 2014) based on the quality control results (FastQC 0.11.9). The high-quality reads were then assembled de novo using SPAdes v4.0.0 (Bankevich et al. 2012). Genome assembly quality was assessed using QUAST v. 5.2.0 (Gurevich et al. 2013), and completeness was evaluated with BUSCO v. 5.7.1.1 (Seppey et al. 2019) against the fungi_odb10.2019-11-20 dataset. Genome annotation was conducted using the Funannotate pipeline v. 1.18.16 (Palmer and Stajich 2020) to predict and annotate gene models within the target genome. Functional annotation was performed using InterProScan v. 5.69-101.0 (Jones et al. 2014) and EggNOG-mapper v. 2.1.9 (Huerta-Cepas et al. 2019). The genome identities were confirmed by comparing extracted ITS barcode sequences.

### ﻿Phylogenetic and barcode analyses

The gene sequences were aligned using MAFFT v. 7.487 (Katoh and Standley 2013) implemented in the CIPRES Science Gateway v. 3.3 (Miller et al. 2010) and manually corrected in BioEdit v. 7.1.8 (Hall 1999), if necessary. Phylogenetic analyses were executed using software packages available on the CIPRES Science Gateway v. 3.3. First, we conducted separate single-marker Maximum likelihood (ML) analyses with RAxML-HPC v. 8.2.12 (Stamatakis 2014). Since we did not detect any conflicting clades between these analyses, the individual sequence alignments were concatenated into two final alignments (deposited in TreeBASE, study number 31694) and subjected to phylogenetic analyses. Partitions, for which we assumed rate heterogeneity, were evaluated using MrModeltest v. 2.4 (Nylander 2004) to determine the best partitioning scheme for our datasets and to select the best-fit models under Akaike information criteria.

In the first phylogenetic analysis of LSU-SSU-*rpb2* sequences, the alignment comprised 107 ingroup strains and included a total of 4 676 characters including gap regions, with 2 452 unique character sites (RAxML). Eighty-six nucleotides (nt) at the beginning of LSU and 70 nt at the beginning of SSU were excluded from the analyses due to the incompleteness of many sequences retrieved from GenBank. Three members of the Savoryellales (Hypocreomycetidae), such as *Bactrodesmiumabruptum*, *Bactrodesmiumdiversum*, and *Neoascotaiwaniaterrestris*, were selected as the outgroup, based on previous research and known relationships within the Sordariomycetes, and availability of molecular data (Réblová et al. 2020). The GTR+I+G best-fit model of nucleotide evolution was selected for each partition.

In the second phylogenetic analysis of ITS-LSU-SSU-*rpb2*-*tef1-α* sequences, the alignment included 11 strains representing 6 species of *Ceratostomella*, encompassing a total of 6 161 characters including gap regions, and 1 099 unique character sites (RAxML). Seventy nucleotides at the beginning of SSU were excluded from the analysis due to the incompleteness of many sequences. The outgroup was selected from members of Chaetosphaeriaceae, specifically *Curvichaetacurvispora* and *Menisporauncinata*, based on known relationships from the first analysis and the availability of DNA sequences. The following best-fit models of nucleotide evolution were selected for each partition: GTR+I for ITS, LSU, *tef1-α*; HKY+I for SSU; and GTR+G for *rpb2*.

The Maximum likelihood analysis was performed with RAxML-HPC v. 8.2.12 with a GTRCAT approximation. Statistical support for the nodes was determined by non-parametric bootstrapping (BS) with 1 000 replicates. The Bayesian Inference (BI) analysis was performed with MrBayes v. 3.2.7 (Ronquist et al. 2012). Two Bayesian searches were conducted using default parameters. The B-MCMCMC (Bayesian-Metropolis-coupled Markov chain Monte Carlo) analyses lasted until the average standard deviation of split frequencies was below 0.01, with trees saved every 1 000 generations with a burn-in of 25%. The BI and ML phylogenetic trees were compared visually in terms of topological conflicts between the supported clades. Nodes supported by values of ≥ 75% ML Bootstrap (BS) and ≥ 0.95 BI Posterior Probability (PP) were deemed well-supported. Phylogenetic trees were visualised in FigTree v. 1.4.3 (Rambaut 2010) and SeaView v. 5.0.5 (Gouy et al. 2010) and edited in Microsoft PowerPoint.

The identity/similarity for ITS, *rpb2* and *tef1-α* sequences of members of *Ceratostomella* was calculated using BioEdit. Histograms of intraspecific and interspecific distances were created for ITS, *rpb2* and *tef1-α* markers to illustrate the extent of overlap for each gene (the so-called barcode gap). A matrix of pairwise distances was computed in MEGA v. 11.0.13 software (Tamura et al. 2021) using the p-distance model with gap deletion (pairwise deletion option) (Kimura 1980). The histograms were plotted in GraphPrism v. 7.03 (Graphpad Software, La Jolla, California) using a bin size of 0.003 (ITS), 0.002 (*rpb2*) and 0.001 (*tef1-α*).

### ﻿Biogeography assessment using published environmental sequences

The biogeography of six out of the eight known *Ceratostomella* species with available ITS sequences was examined following the workflow outlined by Réblová et al. (2022). We utilised the GlobalFungi database (Větrovský et al. 2020), release 5 (16.11.2023), which comprises 84 972 samples from 846 studies, encompassing 593 399 355 unique ITS1 or ITS2 sequences. Since the GlobalFungi database contains separate ITS1 and ITS2 sequences, we analysed these regions independently. To ensure consistency with the ITS spacers stored in GlobalFungi, which were originally extracted using the ITSx extractor (Bengtsson-Palme et al. 2013), we used the ITSx extractor implemented in the SEED2 platform (Větrovský et al. 2018) to extract the spacers from our data. To identify *Ceratostomella* species in GlobalFungi, we conducted an exact hit similarity search in the database with all unique ITS1 and ITS2 haplotypes from our study, searching for published environmental sequences that match in both length and sequence.

## ﻿Results

### ﻿Phylogenetic and barcode analyses

**Figure 1.**
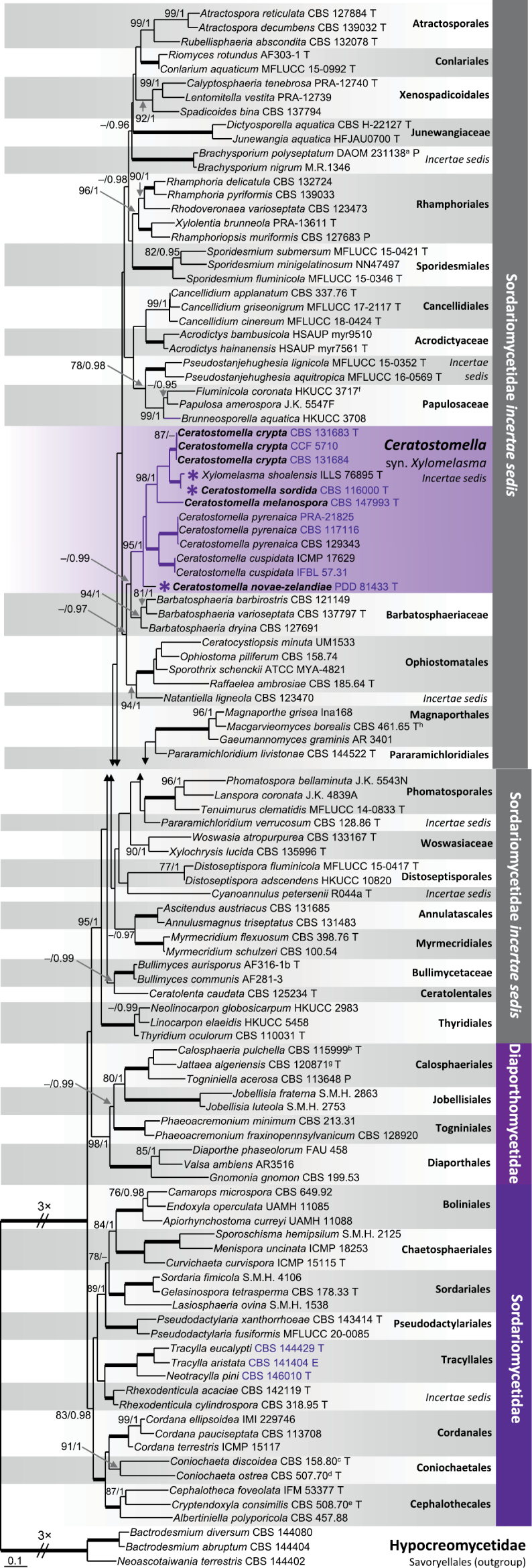
Maximum Likelihood phylogenetic tree of members of the Sordariomycetes based on analysis of LSU, SSU, and *rpb2* DNA sequences. Species names given in bold are taxonomic novelties; the newly acquired strains and those with novel sequences are highlighted in blue font; T, E and P denote ex-holotype, ex-epitype and ex-paratype strains: ^a^ paratype of *Cryptadelphiapolyseptata*, ^b^ holotype of *Calosphaeriophorapulchella*, ^c^ holotype of *Poroconiochaetadiscoidea*, ^d^ holotype of *Coniochaetidiumostreum*, ^e^ holotype of *Cryptendoxylahypophloia*, ^f^ Nom. inval., Art. 36.1(c) (Melbourne), ^g^ holotype of *Jattaeaprunicola*, and ^h^ holotype of *Diplorhinotrichumjuncicola*. Thickened branches indicate branch support with ML BS = 100% and PP values = 1.0. Branch support of nodes ≥ 75% ML and ≥ 0.95 PP is indicated above or below branches. A hyphen (–) indicates values lower than 75% ML BS or 0.95 PP. Purple asterisk before the name indicates former members of the genus *Xylomelasma* within the *Ceratostomella* clade.

In the first phylogenetic analysis, based on a three-gene dataset (LSU-SSU-*rpb2*), we assessed the relationships among *Ceratostomella*, *Xylomelasma*, and representatives of the Sordariomycetes. Phylogenetic trees constructed through BI and ML analyses displayed broad congruence, with the ML tree depicted in Fig. 1. The nodes with support values ≥ 75% ML BS and ≥ 0.95 BI PP were considered well-supported. The phylogram revealed 40 well-supported lineages representing 33 families or orders and seven genera *incertae sedis* belonging to three major, strongly supported clades, i.e. *Diaporthomycetidae* (98% ML BS /1.0 PP), *Sordariomycetidae* (83/0.98) and *Sordariomycetidaeincertaesedis* (95/1.0). Members of *Ceratostomella* and *Xylomelasma* formed a monophyletic, strongly supported and fully resolved clade (95%/1.0) in the *Sordariomycetidaeincertaesedis*. This clade included two strains of *C.cuspidata* (ICMP 17629, IFBL 57.31), three strains of *C.pyrenaica* (CBS 117116, CBS 129343, PRA-21825), the ex-type strains of *X.sordida*CBS 116000 and *X.novae-zelandiae*PDD 81433, and four new isolates initially identified as *X.sordida*. While the two *Ceratostomella* species formed a strongly supported subclade (100/1.0), the original representatives of *Xylomelasma* were not monophyletic. *Xylomelasmasordida* formed a species complex (98/1.0) as a sister to *Ceratostomella*, whereas *X.novae-zelandiae* clustered on a basal branch. These results support the conclusion that both genera are congeneric and that *Xylomelasma* represents a synonym of the older name *Ceratostomella*, with two new combinations proposed, *C.sordida* and *C.novae-zelandiae*. Four new strains acquired in this study represent two cryptic species that are morphologically indistinguishable from *C.sordida*. These strains are described below as *C.crypta* (ex-type strain CBS 131683, CBS 131684, CCF 5710) and *C.melanospora* (ex-type strain CBS 147993). Additionally, *X.shoalensis* holotype ILLS 76895 is shown to be conspecific with *C.sordida*CBS 116000, with their LSU (760 nt long fragment) exhibiting 99.2% sequence similarity. The *Ceratostomella* clade is a sister (62/0.99) to *Barbatosphaeria* (Barbatosphaeriaceae) (94/1.0), though this relationship is not statistically supported in the ML analysis. Other close relatives of *Ceratostomella* include members of the clade (94/1) containing *Ophiostomatales* (100/1) and *Natantiellaligneola*CBS 123470.

**Figure 2.**
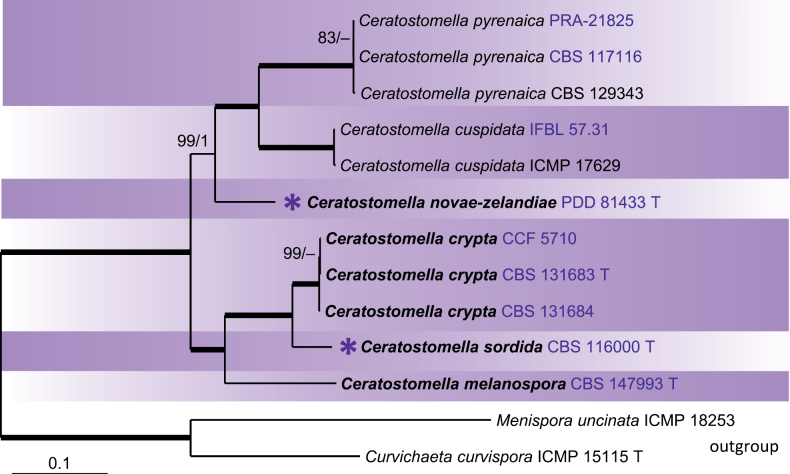
Maximum Likelihood phylogenetic tree of members of *Ceratostomella* based on analysis of ITS, LSU, SSU, *rpb2* and *tef1-α* DNA sequences. Species names given in bold are taxonomic novelties; the newly acquired strains and those with novel sequences are highlighted in blue font. Thickened branches indicate branch support with ML BS = 100% and PP values = 1.0. Branch support of nodes ≥ 75% ML and ≥ 0.95 PP is indicated above or below branches. A hyphen (–) indicates values lower than 75% ML BS or 0.95 PP. Purple asterisk before the name indicates former members of the genus *Xylomelasma* within the *Ceratostomella* clade.

In the second phylogenetic analysis, we utilised a five-gene dataset (ITS-LSU-SSU-*rpb2*-*tef1-α*) to focus on the relationships among *Ceratostomella* species. Both ML and BI trees were congruent, with the ML tree shown in Fig. 2. Compared to the first analysis (Fig. 1), the *Ceratostomella* clade differed in the position of *C.novae-zelandiae*, which appeared on a separate branch as a sister (99/1.0) to the *C.cuspidata* and *C.pyrenaica* subclade (100/1.0). The *C.sordida* species complex consisted of three morphologically indistinguishable species that can be clearly differentiated by molecular data. The low sequence similarity of ITS, *rpb2*, and *tef1-α* markers, along with the high evolutionary divergence among these markers within the *C.sordida* species complex warrant the recognition of three distinct species, including the newly identified *C.crypta* and *C.melanospora*. *Ceratostomellacrypta* was represented by three isolates in the phylogenetic analysis; the strain CBS 131684 exhibited the following sequence similarities with the ex-type strain and the other isolate CCF 5710: 99.6% in ITS, 100% in LSU, SSU, and *tef1-α*, and 99.8% in *rpb2*. *Ceratostomellasordida* and *C.crypta* exhibited sequence similarities of 89.3–90.8% in ITS, 90.4% in *rpb2*, and 96.3% in *tef1-α*. *Ceratostomellasordida* and *C.melanospora* showed similarities of 78% in ITS, 85.2% in *rpb2*, and 96.5% in *tef1-α*. *Ceratostomellacrypta* and *C.melanospora* demonstrated similarities of 70–76.9% in ITS, 84.7–84.9% in *rpb2*, and 93.8% in *tef1-α*.

**Figure 3.**
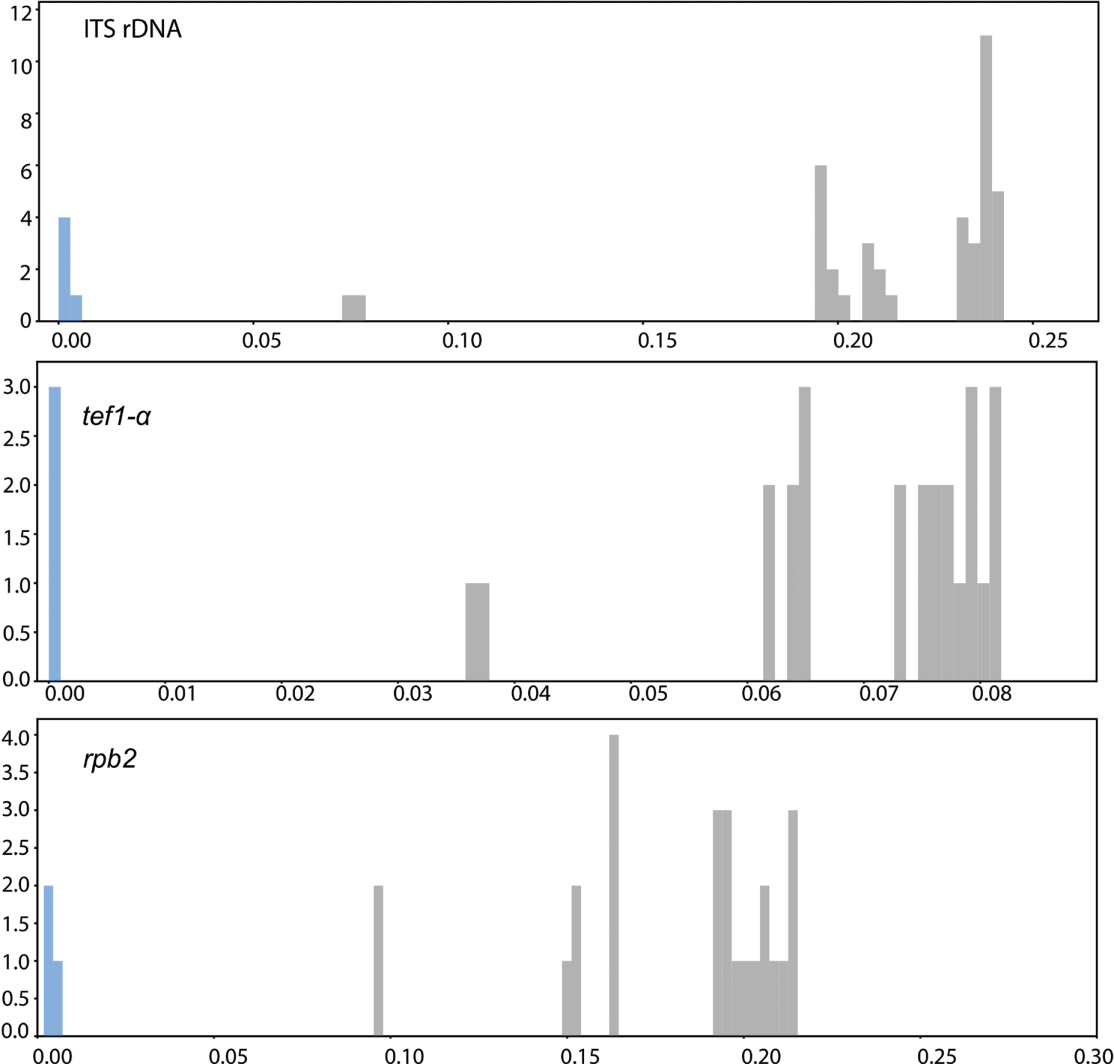
Barcoding gap. The frequency distribution graphs of the Kimura 2-parameter distances of ITS, *rpb2* and *tef1-α* DNA sequences (i.e. barcoding gaps). The intraspecific distances are shown as blue bars and interspecific distances as grey bars.

We recognised a clear barcoding gap in all three markers: ITS, *rpb2* and *tef1-α* (Fig. 3). Regarding species differences in genetic divergence among the three barcodes within the *C.sordida* species complex, the divergence was generally lower between *C.sordida* and *C.crypta* (ITS: 7.5–7.7%, *rpb2*: 9.6%, *tef1-α*: 3.6–3.7%) compared to the higher divergence encountered between *C.sordida* and *C.melanospora* (ITS: 23.7%, *rpb2*: 15%, *tef1-α*: 6.5%). The genetic divergence between the new species, *C.melanospora* and *C.crypta*, ranged from 6.2% in *tef1-α*, 15.1–15.3% in *rpb2* to 23.9% in ITS. The estimates of genetic divergences between *C.melanospora*/*C.sordida* and *C.melanospora*/*C.crypta* in ITS and *rpb2* were the highest within the *C.sordida* species complex. The estimates of evolutionary divergence are provided in the Suppl. material 2.

Whole-genome sequences for the three representative strains were successfully obtained, BioProject: PRJNA1170903. The number of scaffolds ranged from 557 to 9491, with genome completeness, based on conserved fungal genes, between 98.5% and 98.9%. Genome sizes varied from 38.9 to 52.4 Mbp. The annotated genomes have been deposited in the NCBI database, and the quality and completeness of the assembled whole genomes are presented in Table 2.

**Table 2.** Quality and completeness of the obtained whole genome sequences, BioProject: PRJNA1170903.

Strain	Taxon	GenBank biosample numbers	Dataset Complete (BUSCO) (%)	Scaffold N50	Contigs N50	Number of scaffolds	Total length (genome size, bp)
CBS 131683	*C.crypta*	SAMN44110716	98.9	205990	201397	9491	52431894
CBS 147993	*C.melanospora*	SAMN44110894	98.6	315073	299955	1285	38866519
CBS 116000	*C.sordida*	SAMN44113309	98.5	614896	586589	557	49647797

### ﻿Biogeography of *Ceratostomella*

Members of *Ceratostomella* thrive on decaying wood and decaying basidiomata of *Polyporales.* They have also been detected in environmental samples predominantly isolated from air and soil, as well as from deadwood, shoots, roots and water across various habitats. Representatives of the genus *Ceratostomella* are distributed worldwide, primarily in temperate regions of the Northern and Southern Hemispheres, with some occurrences in subtropical (*C.cuspidata*, *C.sordida*) and tropical (*C.sordida*) zones of Asia, Australasia, Europe and North America and South America. According to GlobalFungi, the most common species appears to be *C.sordida*, which is the only species found across various biomes in temperate, subtropical and tropical regions. *Ceratostomellasordida* was identified in 126 samples, compared to other species with known DNA sequences: *C.pyrenaica* (50 samples), *C.cuspidata* (28), *C.crypta* (10), *C.melanospora* (7), and *C.novae-zelandiae* (2). For detailed information, see Suppl. material 3.

### ﻿Taxonomy

#### ﻿ Ceratostomella

Taxon classificationFungiBolinialesBarbatosphaeriaceae

Sacc., Michelia 1: 370. 1878.

B1E140DB-9DA5-5377-A832-1AE226A42AE7

Xylomelasma Réblová, Mycologia 98: 87. 2006. Synonym.

##### Lectotype species.

*Ceratostomellarostrata* (Tode) Sacc., Syll. Fung. 1: 409. 1882 (Lectotype designated by Clements and Shear 1931).

##### Description.

***Sexual morph*.** Ascomata perithecial, non-stromatic, venter globose to subglobose, superficial, semi-immersed or immersed, glabrous, roughened or tuberculate, dark brown to black, usually surrounded by sparse mycelium; hyphae growing out of the sides and bottom of the venter. Necks rostrate, central, cylindrical, straight to slightly flexuous, perpendicular or oblique to almost decumbent toward the substrate, sulcate or glabrous. Ostiolum periphysate. Ascomatal wall leathery to carbonaceous, two-layered. Outer layer consisting of brown, thick-walled cells, textura prismatica to textura angularis to textura epidermoidea, often with a distinct, external crustose layer of heavily pigmented, dark brown cells with opaque walls. Inner layer consisting of thinner-walled, subhyaline to hyaline, elongated and compressed cells. Ascogenous hyphae with croziers, with several lateral and terminal dehiscent cells produced sequentially and simultaneously, from each ascogenous cell one ascus arises as an outgrowth. Paraphyses abundant, unbranched, septate, hyaline, broad-celled and constricted at the septa, wider near the base, tapering, apically free, longer than the asci, disintegrating with age. Asci unitunicate, persistent, clavate to cylindrical-clavate, short-stipitate, truncate to broadly rounded at the apex, tapering toward the base from the sporiferous part, floating freely within the centrum at maturity, with a shallow, sometimes indistinct, non-amyloid apical annulus, 8-spored. Ascus stipe usually contains non-refractive material deposited at the bottom part, visible after ascus dehiscence from the ascogenous cell. Ascospores variable in shape, suballantoid, ellipsoidal to irregularly ellipsoidal, globose or reniform, straight or curved, often flattened on one side, hyaline when young, becoming pale brown to brown before discharge from the ascus, aseptate, smooth, sometimes with indistinct terminal pores at one or both ends, arranged in a fascicle in the upper part of the ascus or 2–3-seriate or uniseriate within the ascus. (Partially adopted from Réblová 2006.) ***Asexual morph*.** Hyphomycetous, dematiaceous; in culture only sterile mycelium was observed.

##### Accepted species.

*Ceratostomellacrypta*, *C.cuspidata*, *C.melanospora*, *C.novae-zelandiae*, *C.pyrenaica*, *C.rhynchophora*, *C.rostrata*, and *C.sordida*.

##### Notes.

Species of *Ceratostomella* exhibit a variety of ascospore shapes, including suballantoid to reniform in *C.cuspidata*, suballantoid non-apiculate in *C.rostrata*, ellipsoidal slightly apiculate in *C.crypta*, *C.melanospora*, *C.rhynchophora*, and *C.sordida*, and ellipsoidal to oblong in *C.pyrenaica*. Réblová (2006) also described *Ceratostomella* sp. M.R. 2592, which has globose ascospores; however, this species lacks molecular data to confirm its placement in the genus. The key to identifying *Ceratostomella* species was provided by Réblová (2006). Table 3 displays morphological characteristics of *Ceratostomella* species accepted in the genus.

**Table 3.** Morphological characteristics of *Ceratostomella* spp.

Taxon	Ascomata size*	Munk pores	Neck	Asci size	Ascospores size	Shape	Sample (GF)**	Ref.
*C.crypta*	350–500	No	sulcate	66–77(–81.5) × 7.5–9.5(–10)	8.5–11 × (4–)4.5–5.5	ellipsoidal, slightly apiculate	10	1
*C.cuspidata*	380–500	Yes	sulcate	21–30 × (5–)6–7	4–5 × 2–3	suballantoid to reniform	28	2
*C.melanospora*	300–480	No	sulcate	63–78 × 6.5–8(–8.5)	(8–)8.5–10.5(–11) × 4–5	ellipsoidal, slightly apiculate	7	1
*C.novae-zelandiae*	310–340	No	smooth	50–60(–65) × 7–8(–9)	7–8 × (3.5–)4–5	ellipsoidal, slightly apiculate	2	2
*C.pyrenaica*	400–550	No	sulcate	(30–)33–40 × 5.5–7	7–9 × 3–4	ellipsoidal to oblong, slightly curved and apiculate	50	2
*C.rhynchophora*	500–650	No	sulcate	(33–)35–44 × 7–8.5(–10)	6–7 × (3.5–)4–5	ellipsoidal, slightly apiculate, sometimes flattened on one side	n/a	2
*C.rostrata*	650–750	Yes	sulcate	(26–)30–39 × 5–6	4.5–6 × 1.5–2	allantoid to suballantoid	n/a	2
*C.sordida*	490–550	Yes	sulcate	(58–)60–76(–81) × 7–10(–13)	9–12 × 4–6	ellipsoidal, slightly apiculate	126	2

* Size of ascomata (diam), asci and ascospores is given in µm. **GF = GlobalFungi database; the number indicates the total number of samples with identical sequences detected in GlobalFungi. n/a = data not available. References: 1 = This study; 2 = Réblová 2006.

#### ﻿ Ceratostomella crypta

Taxon classificationFungiBolinialesBarbatosphaeriaceae

Réblová, Hubka & Jurjević, sp. nov.

94D28FD8-3994-5DE7-BBC4-5F3040D2BCA0

855703

Figs 4 , 5 , 6

##### Etymology.

*Cryptus* (Latin) meaning hidden, secret; referring to cryptic nature of this species, which is morphologically indistinguishable from *C.melanospora* and *C.sordida*.

##### Type.

Czech Republic • South Moravian Region, Břeclav district, obora Soutok near Lanžhot; on decaying deciduous wood; 23 Oct 2004; M. Réblová M.R. 2911 (holotype PRA-21820!, ex-type culture CBS 131683).

##### Description.

***Sexual morph*.** Ascomata non-stromatic, grouped, immersed with only necks protruding, sometimes partially erumpent with bases semi-immersed. Venter 350–500 µm diam, subglobose, upright, dark brown to black, with sparse brown, septate, slightly flexuous hairs 3.5–4.5 µm wide sparsely covering the sides and bottom. Neck 100–120 µm wide, up to 860 µm long, central, cylindrical, upright, tapering at the top, sulcate along the upper half or the whole length. Ostiole periphysate. Ascomatal wall fragile to leathery, 51–72(−82) µm thick, two-layered. Outer layer consisting of thick-walled, dark brown, polyhedral cells with opaque walls of textura prismatica, with several cells forming the external crustose layer ca. 9–14 µm thick, cells tend to be more flattened and paler towards the interior. Inner layer consists of several rows of thin-walled, hyaline, flattened cells. Paraphyses abundant, longer than the asci, becoming partially disintegrated with age, septate, slightly constricted at the septa, hyaline, 5–9.5 µm wide, wider near the base, tapering to ca. 3.5 µm. Asci 66–77(–81.5) × 7.5–9.5(–10) µm (mean ± SD = 74.9 ± 4.4 × 8.7 ± 0.8 μm), 57.5–71.5(–86) µm (mean ± SD = 65.8 ± 2.6 μm) long in the sporiferous part; asci containing mostly collapsed ascospores are generally smaller in size 61–71.5(–74) × 7–8.5 µm (mean ± SD = 66.4 ± 2.9 × 7.8 ± 0.6 μm), 50.5–59 µm (mean ± SD = 54.3 ± 3.5 μm) long in the sporiferous part, broadly rounded to truncate at the apex, cylindrical, with a short tapering stipe, apical annulus non-amyloid, 2.5 µm wide, 1–1.5 µm high, 8-spored. Ascospores 8.5–11 × (4–)4.5–5.5 µm (mean ± SD = 9.5 ± 0.7 × 5 ± 0.3 μm), ellipsoidal, slightly apiculate at both ends, brown, aseptate, smooth, with an inconspicuous germ pore at one or both ends, sometimes with one oil drop, often collapsing, obliquely uniseriate or partially overlapping within the ascus. ***Asexual morph*.** Unknown.

##### Characteristics in culture

**(after 2/4 wk at 23 °C).** On CMD colonies 70–72 mm/mycelium fully covered the plate, circular, flat, margin effuse to fimbriate with a sparse growth, cobwebby, grey-brown, reverse of the same colour. On MLA colonies 50–51 mm diam/mycelium fully covered the plate, margin entire to fimbriate, circular, flat, margin entire, lanose, olivaceous grey, reverse dark olivaceous brown. On OA colonies 83–85 mm diam/mycelium fully covered the plate, circular, margin entire to fimbriate, lanose, olivaceous grey, reverse dark brown. On PCA colonies 62–64 mm diam/mycelium fully covered the plate, circular, flat, margin diffuse, cobwebby, grey-brown, reverse dark brown. Sporulation was absent on all media.

Temperature dependent growth at 30, 35, 37, 41 °C was assessed as colony diam on MEA, PDA, and OA, respectively, after a period of two weeks: 30 °C >90 mm/>90 mm/>90 mm, 35 °C >90 mm/>90 mm/>90 mm, 37 °C >90 mm/>90 mm/>90 mm, 41 °C 46–48 mm/40–41 mm/26–27 mm.

On MCA, colonies are effuse, with commonly submerged subhyaline hyphae that later become vein-like, ranging in colour from brown to dark olivaceous brown, 3–7 µm in diameter, smooth and septate, with occasional tuberose formations. These hyphae often branch to form monilioid hyphae composed of thick-walled cells, varying in shape from nearly rectangular to subglobose. Branching of monilioid hyphae often occurs at right angles, coiling hyphae are also present.

##### Additional specimens examined.

Czech Republic • South Moravian Region: Břeclav district, obora Soutok near Lanžhot; on decaying deciduous wood; 23 Oct 2004; M. Réblová M.R. 2916 (PRA-21821, culture CBS 131684). USA • South Carolina: swab from generating station; Jul 2014; Ž. Jurjević 2471 (culture CCF 5710 = CBS 142809).

##### Habitat and geographical distribution.

Saprobe on decaying deciduous wood in the Czech Republic; it was also isolated from a swab from generating station in the USA (South Carolina). According to GlobalFungi, the species is distributed predominantly in the temperate region of the Northern Hemisphere. Identical sequences were found in 10 samples isolated from air, sediment, soil, and water in various habitats including cropland, forest, shrubland, wetland, anthropogenic, and aquatic environments in the USA (California, Louisiana, North Carolina, and Tennessee).

##### Notes.

Distinguishing *C.crypta* from other species within the *C.sordida* complex presents significant challenges. Nonetheless, *C.crypta* can be reliably differentiated through analysis of ITS, *rpb2*, and *tef1-α* sequences. Moreover, *in vitro* observations revealed that *C.crypta* demonstrates the highest growth rate within its species complex (Fig. 5). It is worth noting that *C.crypta* is unique as its mycelium completely colonizes culture plates within a two-week period when cultivated on MEA and PDA, and within a four-week period when cultivated on CMD, MLA, and PCA media at 23 °C compared to *C.melanospora* and *C.sordida*. In addition, *C.crypta* grows well at 37 °C and exhibits a growth also at 41 °C (Fig. 6). The mycelium of *Ceratostomella* spp. *in vitro* is pigmented and remains sterile. In *C.crypta*, we observed monilioid hyphae, either branched or unbranched, growing from the septate hyphae. On MEA and MCA, monilioid hyphae are more dominant compared to those on PDA. Occasionally, these hyphae appear tuberose (budding-like), a feature that becomes more pronounced with age (Fig. 6F).

**Figure 4.**
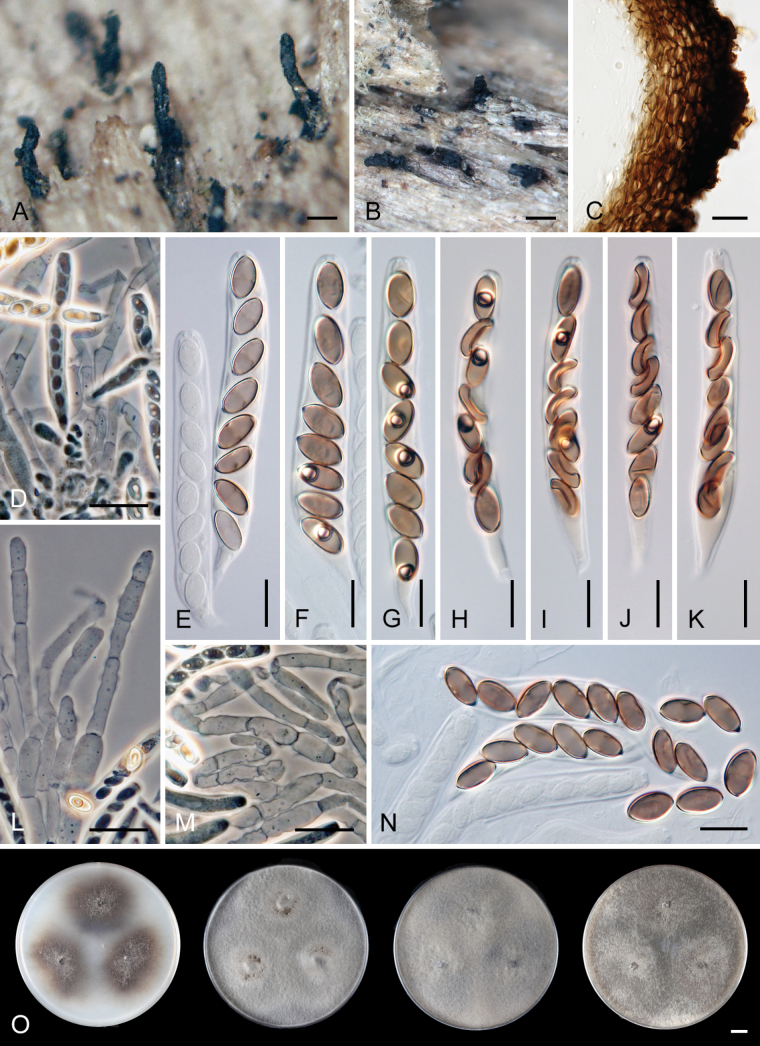
*Ceratostomellacrypta* (**A−N** from holotype PRA-21820 **O** from ex-type strain CBS 131683) **A, B** ascomata **C** a longitudinal section of the ascomatal wall **D** asci with paraphyses and ascogenous cells **E−K** asci with ascospores **L, M** paraphyses **N** ascospores **O** colony morphology at 23 °C after 4 weeks on CMD, MLA, OA and PCA (from left to right). Images: on natural substrate (**A−N**). Scale bars: 500 μm (**A, B**); 20 μm (**C, D, L, M**); 10 μm (**E−K, N**); 1 cm (**O**).

*Ceratostomellacrypta* is represented by three isolates in our phylogeny. Two strains that were isolated from ascospores originate from the same locality in the Czech Republic, while the third is from the USA and is only known in its asexual state. In the Czech specimens, ascospores were observed either strongly collapsed within the asci (CBS 131683, Fig. 4G–K) or mostly retaining their full shape (CBS 131684, Fig. 4E, F) after rehydration, considerably impacting the ascus size. In the closely related species, such as *C.melanospora* and *C.sordida*, the difference in ascus size is less pronounced based on ascospore shape.

**Figure 5.**
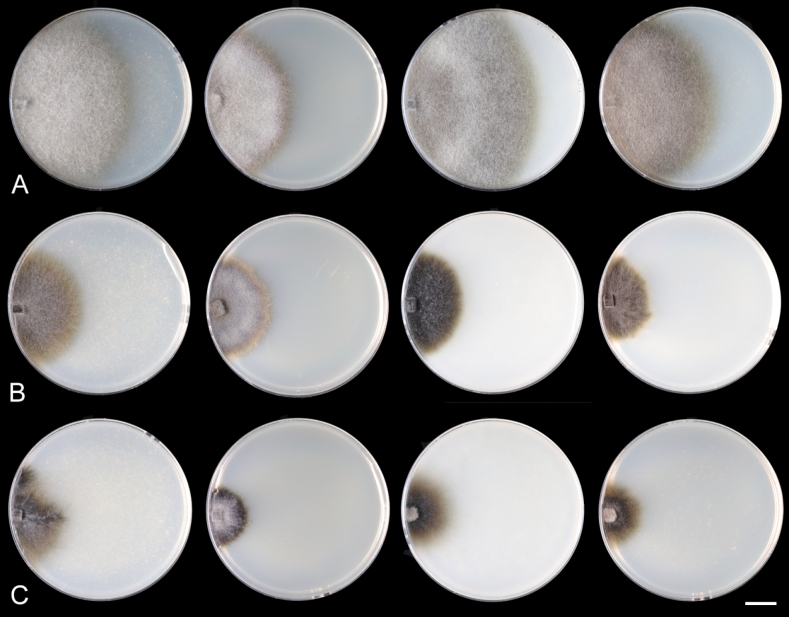
Growth rates *in vitro* of *Ceratostomella* spp. **A***C.crypta*CBS 131683 **B***C.sordida*CBS 116000 **C***C.melanospora*CBS 147993. Colonies on CMD, MLA, OA and PCA (from left to right) after two weeks. Scale bar: 1 cm.

**Figure 6.**
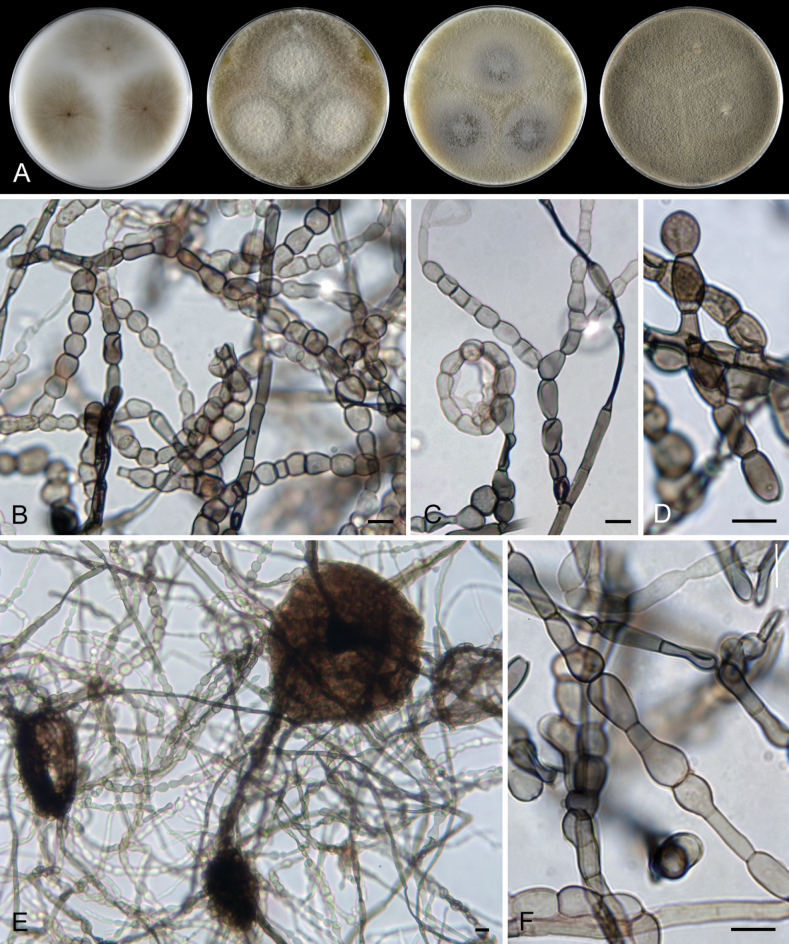
*Ceratostomellacrypta* (CCF 5710) **A** colony morphology at 37 °C after 2 weeks on MCA, MEA, OA and PDA (from left to right) **B, D–F** pigmented monilioid hyphae on CA. **C** monilioid hyphae on MEA. Scale bars: 1 cm (**A**); 10 μm (**B–F**).

#### ﻿ Ceratostomella cuspidata

Taxon classificationFungiBolinialesBarbatosphaeriaceae

(Fr.) Réblová, Mycologia 98: 77. 2006.

14FD68CA-3CC7-5F18-AC76-28215F4B8B80

Sphaeria cuspidata Fr., Syst. Mycol. 2: 220. 1823. Basionym.
≡ Ceratostomacuspidatum (Fr.) Sacc., Syll. Fung. 1: 474. 1882.

##### Description.

See Réblová (2006).

##### Specimens examined.

Belgium • Locality and date unknown; B. Declerque (IFBL 57.31, culture no longer viable). New Zealand • West Coast Region, Westland District, Mount Aspiring National Park, Makarora Bush Walk, 500 m N of NP Headquarters in Makarora; decaying wood of *Nothofagus* sp.; 30 Mar 2005; M. Réblová M.R. 2964/NZ 629 (PDD 123700, culture ICMP 17629).

##### Habitat and geographical distribution.

Saprobe on decaying wood of *Nothofagus* sp., *Quercus* sp., and other unidentified hosts, known in the Czech Republic, New Zealand, Norway and Sweden (Fries 1823; Réblová 2006; MyCoPortal). According to GlobalFungi, *C.cuspidata* is distributed in temperate and subtropical regions in both the Northern and Southern Hemispheres. Identical sequences were found in 28 samples isolated from air and soil in forest and anthropogenic habitats, and occasionally in croplands, grasslands, and shrublands biomes in Australia, Indonesia and New Zealand. The environmental data suggest that *C.cuspidata* is especially widespread in Australasia.

##### Notes.

In our phylogeny, the species is represented by two isolates from Belgium and New Zealand. *Ceratostomellacuspidata* is well distinguishable from other species by its suballantoid to reniform ascospores, often flattened on one side, measuring 4–5 × 2–3 µm (Réblová 2006). The ascospores are arranged in a fascicle or they are 2–3-seriate in the sporiferous part of the ascus. *Ceratostomellarostrata* closely resembles *C.cuspidata* but stands out due to its larger ascomata and narrower allantoid to suballantoid ascospores.

#### ﻿ Ceratostomella melanospora

Taxon classificationFungiBolinialesBarbatosphaeriaceae

Réblová, sp. nov.

F462D576-4D0E-58D6-9E02-BABF396F9FBB

855704

Figs 7 , 8

##### Etymology.

*Melanos* (Greek) meaning black, dark, *spora* (Latin) from Ancient Greek *sporá*, meaning a seed, referring to brown ascospores.

##### Type.

Czech Republic • Pardubice Region, Chrudim district, Železné hory Mts. Protected Landscape Area, Horní Bradlo, Malá Střítež settlement, Polom National Nature Reserve; 600 m alt.; on decaying wood of *Fagussylvatica*; 9 Oct 2020; M. Réblová M.R. 4088 (holotype PRA-21822!, ex-type culture CBS 147993).

##### Description.

***Sexual morph*.** Ascomata non-stromatic, densely grouped or solitary, superficial, semi-immersed or immersed with only neck protruding. Venter 300–480 µm diam, subglobose, upright or lying horizontally in the host tissue, dark brown to black, with brown, septate, slightly flexuous hairs 2.5–4 µm wide sparsely covering the sides and bottom. Neck 90–100 µm wide, up to 500 µm long, central, cylindrical, upright, glabrous, tapering, apex sulcate; the neck is sometimes slightly wider near the top. Ostiole periphysate. Ascomatal wall fragile to leathery, 55–65 µm thick, two-layered. Outer layer consisting of thick-walled, dark brown, polyhedral cells with opaque walls of textura prismatica, with several cells forming the external crustose layer ca. 8–13 µm thick, cells tend to be more flattened and paler towards the interior. Inner layer consists of several rows of thin-walled, hyaline, flattened cells. Paraphyses abundant, longer than the asci, may become partially disintegrated with age, septate, constricted at the septa, hyaline, (5–)6.5–10.5 µm wide, wider near the base, tapering to 3–4 µm. Asci 63–78 × 6.5–8(–8.5) µm (mean ± SD = 70.8 ± 4.2 × 7.2 ± 0.7 μm), 51–60(–62.5) µm (mean ± SD = 57.0 ± 3.3 μm) long in the sporiferous part; truncate at the apex, cylindrical, with a short tapering stipe, apical annulus non-amyloid, ca. 2.5 µm wide, 1–1.5 µm high, 8-spored. Ascospores (8–)8.5–10.5(–11) × 4–5 µm (mean ± SD = 9.3 ± 0.7 × 4.5 ± 0.3 μm), ellipsoidal, slightly apiculate at both ends, brown, aseptate, smooth, with an inconspicuous germ pore at one or both ends, occasionally with one oil drop, often collapsing, obliquely uniseriate or partially overlapping, or partially 2-seriate within the ascus. ***Asexual morph*.** Unknown.

##### Culture characteristics

**(after 2/4 wk at 23 °C).** On CMD colonies 30–32 mm/64–70 mm diam, circular, flat, margin diffuse, cobwebby, mucoid towards the margin, dark brown, with an outer beige zone of conspicuous submerged growth, reverse of the same colour. On MLA colonies 20–21 mm/48–50 mm diam, circular, flat margin fimbriate to somewhat lobate, floccose and whitish grey centrally, cobwebby to mucoid and dark olivaceous grey towards the periphery, reverse of the same colour. On OA colonies 28–30 mm/73–75 mm diam, circular, flat, margin diffuse, lanose and pale olivaceous grey at the centre, sparse to cobwebby and olivaceous black towards the margin, reverse dark brown. On PCA colonies 17–18 mm/53–54 mm diam, circular, flat, margin rhizoidal, submerged, floccose and beige-grey centrally, cobwebby and dark brown towards the margin, reverse dark brown. Sporulation was absent on all media.

Temperature dependent growth at 30, 35, 37, 41 °C was assessed as colony diam on MEA, PDA, and OA, respectively, after a period of two weeks: 30 °C 27–29 mm/23–24 mm/23 mm, 35 °C no growth/no growth/no growth, 37 °C no growth/no growth/no growth, 41 °C no growth/no growth/no growth.

On MLA, colonies are effuse, with submerged hyphae 1–2 μm in diameter. These hyphae are hyaline to subhyaline, sparsely branched, septate, smooth, intertwined with vein-like dark brown hyphae, 3–4.5 μm in diameter. Monilioid hyphae were not observed.

##### Habitat and geographical distribution.

This species is a saprobe on decaying wood of *Fagussylvatica* and is known to occur in the Czech Republic. According to GlobalFungi, identical sequences were identified in seven environmental samples obtained from various localities within the temperate zone of the Northern Hemisphere. These samples were primarily isolated from air and soil in cropland and forest biomes, with occasional findings in anthropogenic habitats in Canada, China, Italy, and Sweden.

**Figure 7.**
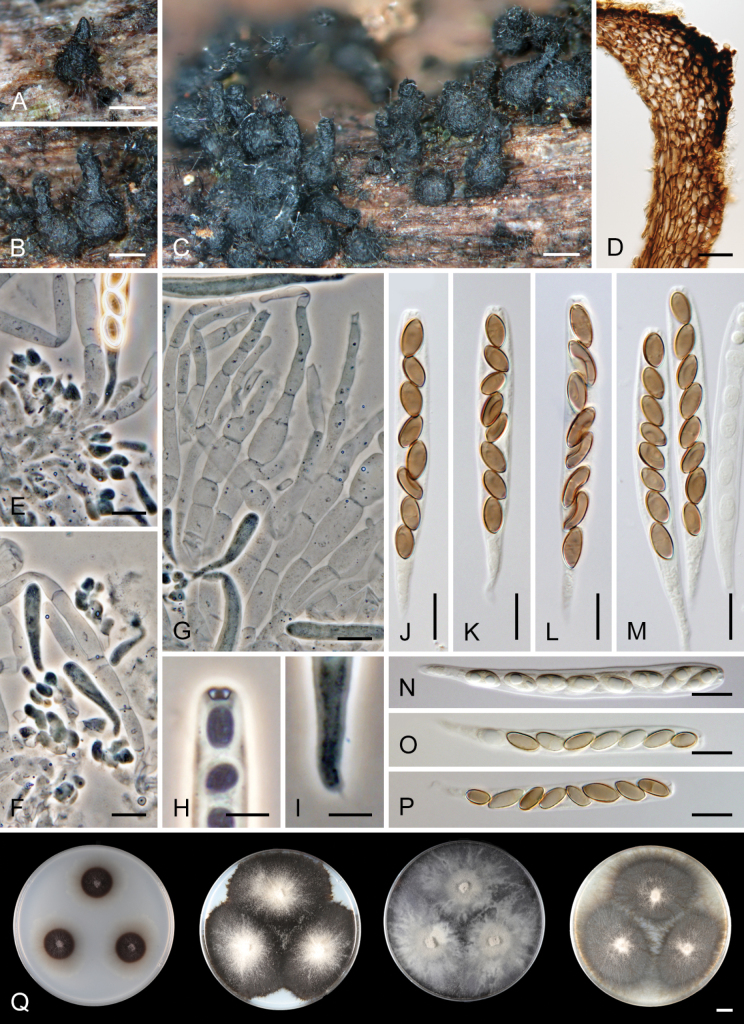
*Ceratostomellamelanospora* (**A–P** from holotype PRA-21822 **Q** from ex-type strain CBS 147993) **A−C** ascomata **D** a longitudinal section of the ascomatal wall **E, F** asci with ascogenous cells **G** paraphyses **H** ascal apex **I** stipe of the ascus **J−M** asci with ascospores **N−O** asci of different ages with maturing ascospores **Q** colony morphology at 23 °C after 4 weeks on CMD, MLA, OA and PCA (from left to right). Images: on natural substrate (**A−P)**. Scale bars: 500 μm (**A−C**); 20 μm (**D**); 10 μm (**E−G, J−P**); 5 μm (**H, I**); 1 cm (**Q**).

##### Notes.

**Figure 8.**
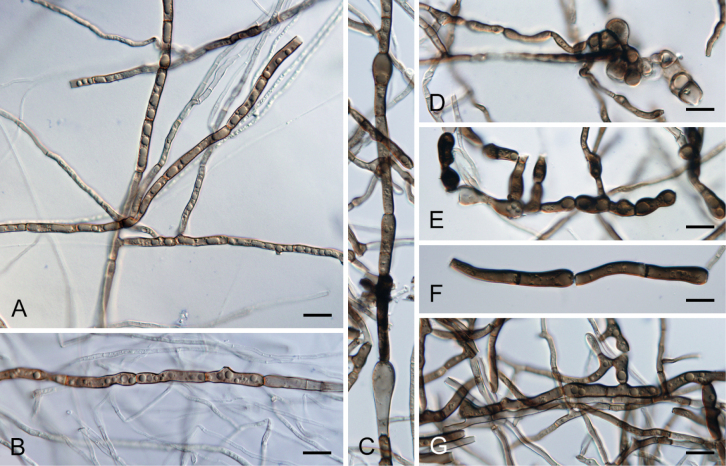
Vegetative mycelium of *Ceratostomella* spp. on MLA **A, B***C.melanospora*CBS 147993 **C−G***C.sordida*CBS 116000. Scale bars: 10 μm (**A−G**).

*Ceratostomellamelanospora* is characterised by ellipsoidal, slightly apiculate, mid-brown ascospores arranged 1-seriately, occasionally partially 2-seriately in the ascus. The species is micromorphologically indistinguishable from *C.crypta* and *C.sordida* but differs by the colony characteristics and can also be clearly differentiated by ITS, *rpb2*, and *tef1-α* sequences. Pigmented monilioid hyphae, which formed abundantly in the culture of *C.crypta* and to some extent in *C.sordida*, were not observed in *C.melanospora* (Fig. 8).

#### ﻿ Ceratostomella novae-zelandiae

Taxon classificationFungiBolinialesBarbatosphaeriaceae

(Réblová) Réblová, comb. nov.

26510240-A801-58DB-B092-62F8593EBD48

855705

Fig. 9

Xylomelasma novae-zelandiae Réblová [as ‘*novaezelandiae*’], Mycologia 98: 87. 2006. Basionym.

##### Description.

See Réblová (2006).

##### Specimen examined.

New Zealand • West Coast Region, Westland District, Haast 300 km SW of Greymouth, Jackson River valley, track to the Lake Ellery; on decaying wood of a stump of *Nothofagus* sp.; 10 Mar 2003; M. Réblová M.R. 2787/NZ 297 (holotype PDD 81433!).

##### Habitat and geographical distribution.

Saprobe occurring on decaying wood of *Nothofagus* sp. in New Zealand (Réblová 2006). According to GlobalFungi, identical sequences were found in two samples isolated from soil in temperate broadleaf forest habitats in New Zealand and Chile.

**Figure 9.**
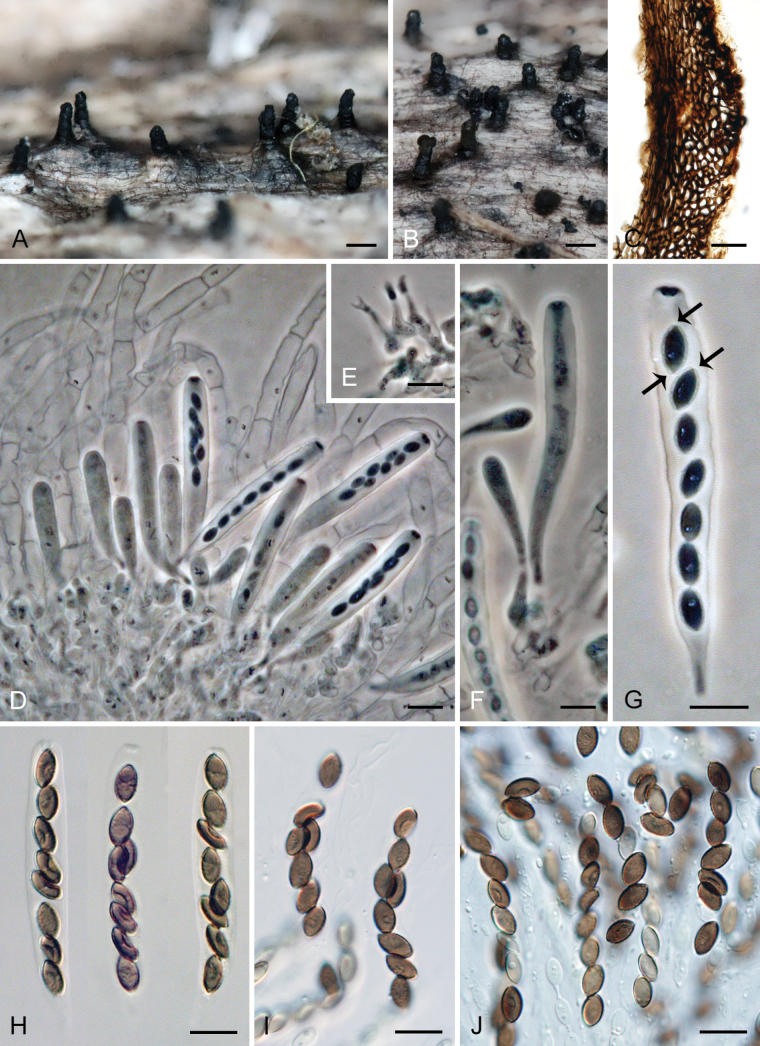
*Ceratostomellanovae-zelandiae* (holotype PDD 81433) **A, B** ascomata **C** a longitudinal section of the ascomatal wall **D–F** paraphyses, ascogenous cells, and asci **G** young ascus (arrows indicate ends of ascospores with pores, where the outer wall becomes thinner) **H–J** asci with ascospores. Images: on natural substrate (**A−J**). Scale bars: 500 μm (**A, B**); 20 μm (**C**); 10 μm (**D−J**).

##### Notes.

*Ceratostomellanovae-zelandiae* is distinguished from the other two species in the *C.sordida* complex by its smaller asci, measuring 50–60(–65) × 7–8(–9) μm, and smaller ascospores, measuring 7–8 × (3.5–)4–5 μm.

#### ﻿ Ceratostomella pyrenaica

Taxon classificationFungiBolinialesBarbatosphaeriaceae

Réblová & J. Fourn., Mycologia 98: 78. 2006.

84DFDD5F-5F89-5A33-91E1-AE9CDBDDA518

##### Description.

See Réblová (2006).

##### Specimens examined.

Czech Republic • South Moravian Region, Hodonín district, Mikulčice, Skařiny Nature Reserve; on decaying wood of a trunk of *Acercampestre*; 24 Oct 2004; M. Réblová, M.R. 2912 (paratype PRA-21823, CBS 117116); • *Ibid*.; Břeclav district, Valtice, Rendez-vous National Nature Monument; on decaying wood of a branch of *Quercus* sp.; 20 Nov. 2010; M. Réblová M.R. 3566 (PRA-21824, CBS 129343); • *Ibid*.; Břeclav district, Milovice, Křivé jezero Nature Reserve, road near Panenský mlýn; on decaying wood of a trunk of *Quercus* sp.; 17 Nov 2010; M. Réblová M.R. 3584 (PRA-21825).

##### Habitat and geographical distribution.

Saprobe on decaying deciduous wood of *Acercampestre*, *Alnusglutinosa*, *Quercus* sp. and other unidentified hosts, and on decaying basidioma of *Trametesgibbosa*, known from the Czech Republic, Belgium and France (Réblová 2006; MyCoPortal; this study). According to GlobalFungi, identical sequences were found in 50 samples collected in temperate and subtropical regions. These samples mainly originated from air, soil, but also shoots, roots and deadwood. *Ceratostomellapyrenaica* is commonly found in croplands and forests, also in grasslands, woodlands and anthropogenic habitats in Croatia, Czech Republic, Italy and the USA (Hawaii, Michigan, and North Carolina).

##### Notes.

*Ceratostomellapyrenaica* is well distinguished from other species by its ellipsoidal to oblong ascospores, which are slightly curved, apiculate at both ends and flattened on one side, pale brown, measuring 7–9 × 3–4 μm.

#### ﻿ Ceratostomella rhynchophora

Taxon classificationFungiBolinialesBarbatosphaeriaceae

(De Not.) Réblová, Mycologia 98: 78. 2006.

76615A6E-1DA3-5BCD-8D2A-4AEE26E1CB4A

Sordaria rhynchophora De Not., Comment. Soc. Crittog. Ital. 2(3): 480. 1867. Basionym.
≡ Ceratostomarhynchophora (De Not.) W. Kirschstein, Krypt.-Fl. Brandenburg 7: 249. 1911.
= Ceratostomanotarisii Sacc., Nuovo Giorn. Bot. Ital. 7: 308. 1875.

##### Description.

See Réblová (2006).

##### Specimens examined.

France • Pyrénés Atlantiques, Ariège, Rimont, Las Muros; on decaying wood of *Prunusdomestica*; 3 Feb 2002; J. Fournier J.F. 02022 (PRA-21826) • *Ibid*.; 21 Apr 2002; J. Fournier J.F. 02070 (PRA-21827).

##### Habitat and geographical distribution.

Saprobe on decaying wood of *Betulapapyrifera*, *Prunusdomestica*, and on decaying basidioma of *Fomesfomentarius*, known in Canada, France, Italy, and Poland (De Notaris 1867; Réblová 2006; MyCoPortal).

##### Notes.

The neotype of this species (Italy, decaying wood of *Prunusdomestica*, P.A. Saccardo, PAD; as *Ceratostomanotarisii*) was designated by Réblová (2006). The species is characterised by ellipsoidal, slightly apiculate ascospores, sometimes flattened on one side, measuring 6–7 × (3.5–)4–5 μm. The ascospores are mid-brown, with a minute pore at each end, and are arranged 1–2-seriately or in a fascicle within the ascus. Given the shape of the ascospores, the species resembles members of the *C.sordida* complex, but it clearly differs by having smaller ascospores.

#### ﻿ Ceratostomella rostrata

Taxon classificationFungiBolinialesBarbatosphaeriaceae

(Tode) Sacc., Syll. Fung. 1: 408. 1882.

422D977E-23E3-5076-BF1A-374FCE1D7878

Sphaeria rostrata Tode, Fungi Mecklenb. Sel. 2:14. 1791. Basionym.
≡ Dryinosphaerarostrata (Tode) Dumort., Comment. bot.: 88. 1822.
≡ Cryptosphaeriarostrata (Tode) Ces. & De Not., Comm. Soc. crittog. Ital. 1(fasc. 4): 231. 1863.
≡ Ceratostomarostratum (Tode) Fuckel, Jahrb. Nassau. Ver. Naturk. 23–24:127. 1870.
≡ Cerastomarostratum (Tode) Quél., Mém. Soc. Émul. Montbéliard, Sér. 2, 5: 521. 1875.
≡ Ceratosphaeriarostrata (Tode) Sacc., Syll. Fung. 2: 227. 1883. (as ‘[Kickx] Sacc.’).
≡ Cerastostomellarostrata (Tode) Massee, Grevillea 17(84): 73. 1889.
≡ Endoxylarostrata (Tode) Munk, Dansk Bot. Ark. 17: 196. 1957.
= Ceratostomagrumsinianum W. Kirschst., Ann. Mycol. 34:199. 1936.
= Wegelinapolyporina M.E. Barr, Cryptogamie, Bryol. Lichenol. 19:170. 1998.^1^

##### Description.

See Réblová (2006).

##### Habitat and geographical distribution.

Saprobe on decaying basidioma of *Fomesfomentarius* and decaying wood of *Acersaccharum*, *Acer* sp., *Coriaria* sp., *Fraxinus* sp., *Morus* sp., *Ostrya* sp., *Quercuspedunculata*, *Quercus* sp., *Populustremuloides*, *Robiniapseudoacacia*, *Ulmusglabra*, *Ulmus* sp., and other unknown hosts, known in Belgium, Canada, Czech Republic, Denmark, Finland, France, Netherlands, Norway, Germany, Poland, Sweden, Switzerland, and the USA (Tode 1791; Kirschstein 1936; Barr 1998; Réblová 2006; MyCoPortal).

##### Notes.

Réblová (2006) designated the lectotype (illustration; Tode 1791: fig. 79) and epitype (Fries´s Scleromyceti Sueciae 116, decayed wood, PRM 666367) of *C.rostrata*. Untereiner (1993), in her revision of the genus *Endoxyla*, cited *Ceratostomellaampullasca* (Saccardo 1882) and *Endoxylalaevirostris* (Munk 1965) as synonyms of *C.rostrata*. However, recent collections and molecular DNA data have revealed that these two species are conspecific and were reclassified as *Natantiellaligneola* (Réblová and Štěpánek 2009). There are numerous records of *C.rostrata* in MyCoPortal; however, these may represent species of *Endoxyla*, as the synonymy of this species was only recently clarified. Accurate identification would require a thorough examination of the herbarium specimens cited, which are housed in various collections around the world.

*Ceratostomellarostrata* is somewhat similar to *C.cuspidata*; however, it differs in having larger ascomata and pale brown, allantoid to suballantoid, narrower ascospores measuring 4.5–6 × 1.5–2 μm. These ascospores are typically arranged in a fascicle in the upper part of the ascus or are 2–3-seriate within the ascus. Molecular data for this species are not available.

#### ﻿ Ceratostomella sordida

Taxon classificationFungiBolinialesBarbatosphaeriaceae

(Réblová) Réblová, comb. nov.

9EAC725B-4F60-58E2-8128-5554A8A8C095

855706

Figs 8 , 10

Xylomelasma sordida Réblová, Mycologia 98: 88. 2006. Basionym.

##### Description.

See Réblová (2006).

##### Characteristics in culture

**(after 2/4 wk at 23 °C).** On CMD colonies 38–40 mm/72–73 mm diam, circular, flat, margin diffuse to slightly fimbriate, cobwebby, olivaceous-brown, reverse of the same colour. On MLA colonies 35–36 mm/76–80 mm diam, circular, flat, sub-entire with a tendency towards a fimbriate edge, lanose, zonate, whitish grey centrally with an olivaceous brown intermediate zone, dark olivaceous grey towards the periphery, reverse dark olivaceous. On OA colonies 34–35 mm/77–79 mm diam, flat, margin diffuse, floccose to cobwebby, olivaceous grey to olivaceous brown, aerial hyphae with numerous colourless droplets, reverse of the same colour. On PCA colonies 30–31 mm/58–60 mm diam, circular, flat, margin rhizoidal, sparse to cobwebby, whitish brown at the centre, dark brown towards the periphery, reverse dark brown. Sporulation was absent on all media.

**Figure 10.**
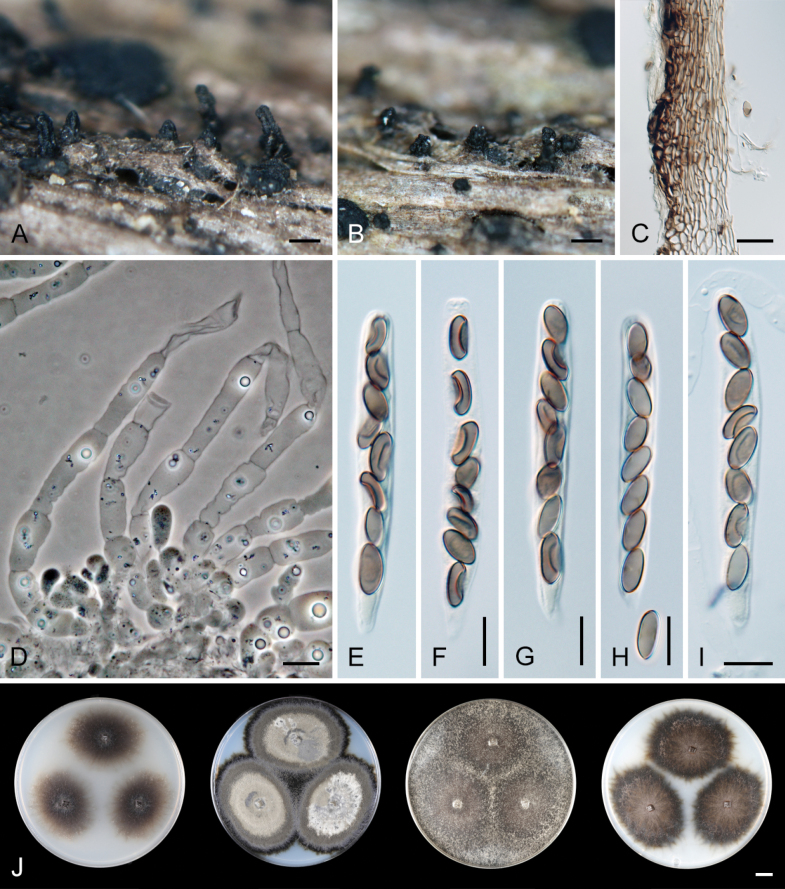
*Ceratostomellasordida* (**A−I** from holotype PRM 902275 **J** from ex-type strain CBS 116000) **A, B** ascomata **C** a longitudinal section of the ascomatal wall **D** paraphyses with ascogenous cells **E−I** asci with ascospores **J** colony morphology at 23 °C after 4 weeks on CMD, MLA, OA and PCA (from left to right). Images: on natural substrate (**A−I**). Scale bars: 500 μm (**A, B**); 20 μm (**C**); 10 μm (**D−I**); 1 cm (**J**).

Temperature dependent growth at 30, 35, 37, 41 °C was assessed as colony diam on MEA, PDA, and OA, respectively, after a period of two weeks: 30 °C 58–60 mm/55–58 mm/49–50 mm, 35 °C 60–61 mm/58–59 mm/46–47 mm, 37 °C 37–39 mm/30 mm/14–19 mm, 41 °C germination only/ 5–7 mm/no growth.

On MLA, colonies are effuse, with submerged hyphae 1.5–3 μm in diameter; hyphae are smooth, branched, septate, subhyaline to pale brown, intertwined with dark brown, vein-like hyphae with occasional tuberose formations, 4–6.5 μm in diameter. Dark brown monilioid hyphae 5.5–9 μm in diameter, composed primarily of rectangular cells, occur rarely.

##### Specimen examined.

France • Pyrénés Atlantiques: Ariège, Lescure, Bois du Pas du Baup; 500 m alt.; on rotten wood of *Alnusglutinosa*; 24 Feb 2004; J. Fournier J.F. 04020 (holotype PRM 902275!, ex-type culture CBS 116000).

##### Habitat and geographical distribution.

Saprobe that decomposes the wood of *Alnusglutinosa*, *Eucalyptusviminalis*, *Fagussylvatica*, *Populus* sp. and other unidentified hosts. It has been found in Argentina, Canada, Czech Republic, Denmark, France, Hungary, New Zealand and Norway (Réblová 2006; MyCoPortal). According to GlobalFungi, *C.sordida* is distributed worldwide in temperate, subtropical and tropical regions of Asia, Australasia, Europe and North and South America. Identical sequences were found in 126 samples isolated mainly in cropland and forest habitats, but also in air, dust, deadwood, grassland, roots, shoots, soil (including rhizosphere soil), tundra, water, and aquatic and anthropogenic habitats in Argentina, Austria, Brazil, Canada, China, Costa Rica, France, French Guyana, Italy, Indonesia, Japan, New Zealand, Papua New Guinea, Romania, Russia, Spain, Sweden, Switzerland, South Korea, and the USA (California, Florida, Iowa, Louisiana, North Carolina, Oklahoma, Pennsylvania, and Tennessee).

##### Notes.

In culture, *C.sordida* rarely forms short monilioid hyphae (Fig. 8), in contrast to *C.crypta*, where these hyphae form frequently and are much longer (Fig. 6). We re-examined the holotype of *C.sordida* (Réblová 2006), focusing on the measurements of both asci and ascospores. Despite fully rehydrating the centrum of the aged material, we observed that the ascospores were slightly smaller 8–10.5 × (3.5–)4–5 µm compared to the previously recorded dimensions of 9–12 × 4–6 µm. Similarly, the asci showed a narrower width of 6.5–8 µm, contrasting with the previously reported range of 7–10(–13) µm. For accuracy, we provide both measurements in the fresh and aged material. Two species introduced in this study, *C.crypta* (CBS 131683, CBS 131684) and *C.melanospora* (CBS 147993), were hidden among the herbarium material labelled as *C.sordida*.

### ﻿Excluded or ambiguous species

#### ﻿ Xylomelasma moderata

Taxon classificationFungiBolinialesBarbatosphaeriaceae

Lar.N. Vassiljeva & S.L. Stephenson, Mycosphere 5: 223. 2014. Nom. inval., Art. F.5.1 (Shenzhen).

13AA519B-5F37-55B7-AE14-2618BE7CFE7F

##### Notes.

The species was collected on unidentified decaying wood in the USA (Virginia) (Vassiljeva and Stephenson 2014). However, it was not validly published as an identifier issued by a recognised repository was not cited in the protologue. The species is morphologically distinct from *Ceratostomella* and, based on available morphological data, it represents *Calyptosphaeriasubdenudata* (Réblová et al. 2018).

#### ﻿ Xylomelasma shoalensis

Taxon classificationFungiBolinialesBarbatosphaeriaceae

A.N. Mill., Y. Marín & Stchigel, Sydowia 68: 224. 2016.

08CF5D47-1FDE-5560-B9F8-929D554426BA

##### Specimen examined.

USA – Illinois • Montgomery County, Shoal Creek Conservation Area; 39.1871, -89.5963; on 6 cm. diam. decorticated branch on the ground; 4 Apr 2004; A.N. Miller ANM 1 (holotype ILLS 76895!).

##### Notes.

The only available LSU sequence (KX290919, Hernández-Restrepo et al. 2016) of *X.shoalensis* indicates that the species is a member of *Ceratostomella* (Fig. 1). Based on the sequence similarity with *X.sordida* (99.2%), they are likely conspecific. However, according to its diagnosis, this species does not match the generic delimitation of *Ceratostomella*. *Xylomelasmashoalensis* was described with immersed, globose to subglobose and smaller (175–265 μm diam) ascomata featuring a rostrate, slender, non-sulcate neck and hyaline to yellowish-brown, oblong to suballantoid, septate ascospores in unitunicate asci. Its LSU sequence was not obtained from a mycelium of an axenic culture but directly from ascomata on the host. It is evident that morphology belongs to a different genus and species. We examined the holotype, but no traces of a *Ceratostomella*-like fungus could be found. Therefore, due to this ambiguity, *X.shoalensis* is excluded from *Ceratostomella*.

## ﻿Discussion

Phylogenetic analyses utilising three and five molecular markers, respectively, revealed that *Ceratostomella* (Saccardo 1878) and *Xylomelasma* (Réblová 2006) are congeneric. The analysed species included *C.cuspidata* and *C.pyrenaica* representing *Ceratostomella*, along with the ex-type strains of *X.sordida*, the type of *Xylomelasma*, and *X.novae-zelandiae*. In both phylogenies, *Ceratostomella* consisted of three subclades: *Ceratostomella*, the *C.sordida* species complex, and *C.novae-zelandiae*. However, the position of *C.novae-zelandiae* varied between the two data sets; it either clustered on a basal branch or as a sister to the *Ceratostomella* subclade. *Ceratostomellanovae-zelandiae* was originally placed in *Xylomelasma* based on morphological similarities, as no living culture or DNA data were available at the time (Réblová 2006). In this study, DNA was successfully extracted from ascomata of the holotype of *X.novae-zelandiae*PDD 81433 and new ITS, LSU, and SSU sequences were generated. However, the amplification of protein-coding genes was not successful. *Ceratostomella* is currently recognised with eight species which are listed here. Another species, referred to as *Ceratostomella* sp., was placed in the genus by Réblová (2006). It is characterised by its unique globose ascospores, which are distinct from the reported ascospore variability. This species has not been formally described due to the limited herbarium material that could serve as a holotype and lack of a living culture. Recollection and molecular data of this species are needed to support its placement in the genus.

*Ceratostomella* and *Xylomelasma* exhibit high morphological similarity, with differences primarily in the position of ascospores within the ascus, the variable visibility of the apical annulus, the ornamentation of the neck, and the morphology of the paraphyses. However, the phylogenetic analysis revealed that *Ceratostomella* exhibits greater variability in these morphological traits than previously recognised, supporting the reclassification of *Xylomelasma* as a synonym of *Ceratostomella*. Smaller ascospores, ranging from suballantoid to reniform shapes, tend to be arranged 2–3-seriately or form fascicles in the sporiferous part of the ascus, whereas ellipsoidal and globose ascospores are generally 1–2-seriate within the ascus. The ascospores are aseptate, glabrous and hyaline when young, becoming brown at maturity before being released from the asci. Characters such as the terminal germination pores are particularly well-visible in immature hyaline ascospores (Fig. 9G). A common characteristic of *Ceratostomella* ascospores is their frequent collapsing apparently upon drying, which may influence the size of the sporiferous part of the ascus.

Species of *Ceratostomella* typically possess a thick ascomatal wall, often adorned with tubercles on the exterior. This wall is composed of thick-walled, dark brown to dark reddish-brown cells, which may contain Munk pores (*C.cuspidata*, *C.rostrata*, and *C.sordida*). The neck in all species is sulcate and ornamented with 4–5 ridges at the top, except for *C.novae-zelandiae*, which has a glabrous neck. The apical, non-amyloid annulus is present in all species and is most visible with phase contrast microscopy, although its visibility can vary. In the former *Xylomelasma* species, *C.sordida* and *C.novae-zelandiae*, the paraphyses are composed of slightly longer cells, but are similarly constricted, primarily in the lower part.

A prominent morphological trait shared by both genera is the ascogenous system. This system comprises short ascogenous hyphae with lateral and terminal discrete cells from which asci emerge as outgrowths. The asci and ascogenous cells are connected by a tapering stipe; its bottom part is sometimes visible as a thread-like connective between the ascus and ascogenous cell. The stipe eventually disintegrates at maturity, allowing the asci to float freely in the centrum. The ascus stipe often contains non-refractive material deposited at the basal part, which becomes visible after the ascus dehisces from the ascogenous cell.

The morphology of the ascogenous system can be peculiar in some taxa and has significant diagnostic value at the genus level. These taxonomically important traits include the attachment of asci to ascogenous hyphae, the presence or absence of discrete cells from which asci arise, and the overall architecture of these formations. For instance, members of the order Calosphaeriales, many of which include former *Ceratostomella* species, (including genera such as *Calosphaeria*, *Flabellascus*, *Jattaea*, *Pleurostoma*, and *Togniniella*) and Togniniales (*Phaeoacremonium*) possess morphologically distinct ascoma centrums specific to each genus (Barr 1985; Barr et al. 1993; Mostert et al. 2006; Réblová 2011; Réblová et al. 2015a).

Another example of a distinct ascogenous apparatus is found in the genus *Barbatosphaeria* of Barbatosphaeriaceae, which encompasses several species initially classified in *Ceratostomella*. This feature was first observed in *B.fagi* by Samuels and Candoussau (1996) and later recognised as a diagnostic feature of the genus by Réblová et al. (2015b). In *Barbatosphaeria*, the asci taper towards a slender stipe, with the basal part of the stipe conspicuously swollen. This swollen base remains attached to the ascogenous hyphae after the mature ascus is liberated and floats freely in the centrum. The attachment of asci to ascogenous hyphae and the mechanism of ascus dehiscence in *Barbatosphaeria* is somewhat similar to that of *Ceratostomella*, its closest relative, although the stipe in *Barbatosphaeria* is more robust and does not transform into a thread-like filament. The ascogenous cells present in *Ceratostomella* are absent in *Barbatosphaeria*. However, the attached torso of the swollen base of the ascus stipe in *Barbatosphaeria* mimics these cells and may represent an evolutionary pathway, leading to the development of discrete ascogenous cells.

Phylogenetic analyses using three distinct barcodes have uncovered two cryptic species within the *C.sordida* complex, now identified as *C.crypta* and *C.melanospora*. By analysing five genes: the slow-evolving rDNA genes LSU and SSU, alongside a rapidly evolving ITS gene (primary fungal barcode, Schoch et al. 2012) and the slow-evolving protein-coding genes *rpb2* and *tef1-α* (non-rDNA secondary barcodes, Stielow et al. 2015), we were able to elucidate the phylogenetic relationships within the *C.sordida* species complex (Fig. 2). These findings were corroborated by phylogenetic analysis based on the LSU, SSU, and *rpb2* genes (Fig. 1), further validating the distinctiveness of the new species within the complex.

Traditional diagnostic morphological characters have proven insufficient for characterising species within *C.sordida* complex. *Ceratostomellacrypta* and *C.melanospora* are morphologically indistinguishable from *C.sordida* and from each other in terms of ascospores, asci, paraphyses, and ascomata, but they can be clearly differentiated by molecular data and the size of their genome. In culture, their vegetative mycelium was darkly pigmented and fast-growing, with all species remaining sterile on various nutrient media and when exposed to UV light. Although no significant morphological differences could be identified among members of the *C.sordida* species complex on the natural substrate, *C.crypta* typically formed monilioid hyphae (Fig. 6) and demonstrated the fastest growth rate *in vitro* (Fig. 5). Notably, within the same time frame, the mycelium of *C.crypta* covered the entire plate (or nearly the entire plate) when grown on MEA, OA and PDA (in two weeks) and CMD, MLA, OA and PCA (in four weeks) compared to *C.melanospora* and *C.sordida*. Interestingly, *C.crypta* and *C.sordida* exhibited growth at 37 °C, which is one of the four key criteria for a fungal strategy to colonize and parasitize the tissues of humans and other mammals (Köhler et al. 2015). Additionally, *C.crypta* demonstrated growth at 41 °C on MEA, OA, and PDA, whereas *C.sordida* exhibited growth at this temperature only on PDA.

The closest relative to *Ceratostomella* recruits from *Barbatosphaeria*. *Ceratostomella* and *Barbatosphaeria* form sister clades; however, their relationship is not statistically supported (–/0.99). This grouping (–/1.0) was first identified in the molecular systematic study of *Barbatosphaeria*, where it received strong support in BI analysis but no statistical support in ML analysis (Réblová et al. 2015b). Zhang et al. (2017) proposed the family Barbatosphaeriaceae to include *Barbatosphaeria*, *Ceratostomella* and *Xylomelasma*), although the grouping was supported only in the BI analysis. The lack of statistical support from ML analysis for this monophyly does not warrant the inclusion of *Ceratostomella* in Barbatosphaeriaceae, as proposed by Zhang et al. (2017). Currently, *Ceratostomella* is accepted as a genus *incertae sedis*, while Barbatosphaeriaceae remains a monotypic family. We suggest that better taxon sampling of species, currently unknown to us, is needed to provide support for the inclusion of *Ceratostomella* in Barbatosphaeriaceae.

*Ceratostomella* shares several morphological similarities with the genus *Melanospora* (Corda 1837) of the family Ceratostomataceae (*Melanosporales*, *Hypocreomycetidae*), such as ascomata with a rostrate neck (which may be adorned with coronal setae or lack them) and dull brown, aseptate ascospores with terminal pores. However, *Melanospora* distinctly differs from *Ceratostomella* by its evanescent asci, the absence of paraphyses, and the presence of pseudoparenchyma in the centrum (Luttrell 1951; Cannon and Hawksworth 1982). *Ceratostomella* also exhibits certain similarities to the genus *Cannonia* (Taylor and Hyde 1999), including dark ascomata with long necks and brown ellipsoidal ascospores. Nonetheless, *Cannonia* can be distinguished from *Ceratostomella* by its ascospores with a full-length germ slit, filiform paraphyses, the presence of a rudimentary stroma, and a dark clypeus around the base of the neck. Additionally, *Ceratostomella* differs from both genera in the morphology of its ascogenous system.

## ﻿Conclusions

This study provides new morphological, molecular, and biogeographical data, offering deeper insights into the genus *Ceratostomella* and clarifying interspecific relationships. Based on phylogenetic analyses and comparative morphological studies, we have transferred the genus *Xylomelasma* to *Ceratostomella*, proposed two new combinations, and described two new species. We recognise eight species within the genus. Members of *Ceratostomella* are distributed worldwide in temperate, subtropical and tropical zones of Asia, Australasia, Europe and North and South America. Based on field observations (Réblová 2006; this study) and metabarcoding data in GlobalFungi (Větrovský et al. 2020), *C.sordida* emerges as the most common species of *Ceratostomella* among those with available DNA sequence data. It was identified in 126 environmental samples and several herbarium collections. On the other hand, *C.novae-zelandiae* is recognised as a rare species. It has been identified in only one field record from New Zealand and two environmental samples from GlobalFungi (Chile and New Zealand), confirming the occurrence of this species in the Southern Hemisphere. To improve the standards and reproducibility of our research, we are publishing WGS data. This not only supports future descriptions of new ‘dark taxa’ from environmental DNA samples but also facilitates the classification of *Ceratostomella* diversity. By characterising all representatives of the genus through genomic data, future taxonomic efforts will be more streamlined and accurate.

Despite these advances, information on the asexual morph remains lacking, as it did not form in any of the analysed species (Réblová 2006; this study). We advocate the use of dual barcoding, particularly employing ITS primary barcode and secondary barcodes such as *tef1-α* and *rpb2*, to differentiate among members of *Ceratostomella*, especially among morphologically indistinguishable species. Additionally, further research focusing on comprehensive taxon sampling and exploring environmental DNA could enhance our understanding of the diversity and ecological roles of these fungi.

## Supplementary Material

XML Treatment for Ceratostomella

XML Treatment for Ceratostomella crypta

XML Treatment for Ceratostomella cuspidata

XML Treatment for Ceratostomella melanospora

XML Treatment for Ceratostomella novae-zelandiae

XML Treatment for Ceratostomella pyrenaica

XML Treatment for Ceratostomella rhynchophora

XML Treatment for Ceratostomella rostrata

XML Treatment for Ceratostomella sordida

XML Treatment for Xylomelasma moderata

XML Treatment for Xylomelasma shoalensis

## Footnotes

Synonymy adopted from Réblová (2006).

## ﻿Additional information

### Author ORCIDs

Martina Réblová https://orcid.org/0000-0001-5229-1709

Jana Nekvindová https://orcid.org/0000-0002-2861-5483

Miroslav Kolařík https://orcid.org/0000-0003-4016-0335

Željko Jurjević https://orcid.org/0000-0002-5556-4722

Michal Kolář https://orcid.org/0000-0002-4593-1525

Vít Hubka https://orcid.org/0000-0003-4583-6496

### Data availability

All of the data that support the findings of this study are available in the main text or Supplementary Information.

## Supplementary materials

Supplementary material 1 Taxa, isolate information and sequences retrieved from GenBank Data type pdf https://binary.pensoft.net/file/1179949This dataset is made available under the Open Database License (http://opendatacommons.org/licenses/odbl/1.0/). The Open Database License (ODbL) is a license agreement intended to allow users to freely share, modify, and use this Dataset while maintaining this same freedom for others, provided that the original source and author(s) are credited.Author: Martina Réblová

Supplementary material 2 Estimates of evolutionary divergence between ITS rDNA, *rpb2* and *tef1-α* sequences Data type pdf https://binary.pensoft.net/file/1179950This dataset is made available under the Open Database License (http://opendatacommons.org/licenses/odbl/1.0/). The Open Database License (ODbL) is a license agreement intended to allow users to freely share, modify, and use this Dataset while maintaining this same freedom for others, provided that the original source and author(s) are credited.Author: Miroslav Kolařík

Supplementary material 3 Biogeographical distribution, substrate, habitat and other detailed metadata for *Ceratostomella* species with available ITS sequences inferred from the GlobalFungi database Data type pdf https://binary.pensoft.net/file/1179951This dataset is made available under the Open Database License (http://opendatacommons.org/licenses/odbl/1.0/). The Open Database License (ODbL) is a license agreement intended to allow users to freely share, modify, and use this Dataset while maintaining this same freedom for others, provided that the original source and author(s) are credited.Author: Martina Réblová
