# Supplementary material for: ﻿Re-evaluation of Ceratostomella and Xylomelasma with introduction of two new species (Sordariomycetes)
Source: MycoKeys. 2024 Nov 21;110:319–60. doi: 10.3897/mycokeys.110.136844 (PMC11605300; doi:10.3897/mycokeys.110.136844)
Supplement: Supplementary material 1 — Taxa, isolate information and sequences retrieved from GenBank [file mycokeys-110-319-s001.pdf]

Supplementary Table S1. Taxa, isolate information and sequences retrieved from GenBank.

Taxon	Source	Status*	GenBank accession numbers		
			nucLSU	nucSSU	*rpb2*
Acrodictys bambusicola	HSAUP myr9510		KX033564	KX033535	—
Acrodictys hainanensis	HSAUP myr7561	T	KX033565	KX033536	—
Albertiniella polyporicola	CBS 457.88		AF096185	AF096170	LT634061
Annulusmagnus triseptatus	CBS 131483		GQ996540	JQ429242	JQ429258
Apiorhynchostoma curreyi	UAMH 11088		JX460989	KY931894	KY931926
Ascitendus austriacus	CBS 131685		GQ996539	GQ996542	JQ429257
Atractospora decumbens	CBS 139032	T	KT991658	KT991640	KT991647
Atractospora reticulata	CBS 127884	T	KT991660	—	KT991649
Bactrodesmium abruptum	CBS 144404		MN699408	MN699365	MN704288
Bactrodesmium diversum	CBS 144080		MN699415	MN699371	MN704294
Barbatosphaeria barbirostris	CBS 121149		EF577059	KM492851	KM492903
Barbatosphaeria dryina	CBS 127691		KM492864	KM492852	KM492904
Barbatosphaeria varioseptata	CBS 137797	T	KM492869	KM492857	KM492907
Brachysporium nigrum	M.R. 1346		KT991662	KT991643	KT991652
Brachysporium polyseptatum	DAOM 231136[a]	P	AY281102	—	—
Brunneosporella aquatica	HKUCC 3708		AF132326	—	—
Bullimyces aurisporus	AF316-1b	T	JF775590	JF758614	—
Bullimyces communis	AF281-3		JF775585	JF758617	—
Calosphaeria pulchella	CBS 115999[b]	T	AY761075	AY761071	GU180661
Calyptosphaeria tenebrosa	PRA-12740	T	KY931810	KY931865	KY931837
Camarops microspora	CBS 649.92		AY083821	DQ471036	DQ470937
Cancellidium applanatum	CBS 337.76	T	MH872755	—	—
Cancellidium cinereum	MFLUCC 18-0424	T	MT370363	MT370351	MT370486
Cancellidium griseonigrum	MFLUCC 17-2117	T	MT370364	MT370352	MT370487
Curvichaeta curvispora	ICMP 15115	T	GU180636	AY502933	GU180655
Cephalotheca foveolata	IFM 53377	T	AB178269	AB278171	LT634043
Ceratocystiopsis minuta	UM1533, WIN(M)1537		EU913657	HQ634854	—
Ceratolenta caudata	CBS 125234	T	JX066704	JX066708	JX066699
Coniochaeta discoidea	CBS 158.80[c], SANK 12878	T	MH873023	AJ875179	AY780191
Coniochaeta ostrea	CBS 507.70[d]	T	DQ470959	DQ471007	DQ470909
Conlarium aquaticum	MFLUCC 15-0992	T	MF374363	MF374372	—
Cordana ellipsoidea	IMI 229746		HE672156	—	—
Cordana pauciseptata	CBS 113708		EF204507	—	EF204490
Cordana terrestris	ICMP 15117		EF063573	—	—
Cryptendoxyla consimilis	CBS 508.70[e]	T	LT633941	LT633942	LT634048
Cyanoannulus petersenii	R044a	T	AY316358	—	—
Diaporthe phaseolorum	FAU 458, NRRL 13736		U47830	L36985	AY641036
Dictyosporella aquatica	CBS H-22127	T	KT241022	KT241023	—
Distoseptispora adscendens	HKUCC 10820		DQ408561	—	DQ435092
Distoseptispora fluminicola	MFLUCC 15-0417	T	KU376270	—	—
Endoxyla operculata	UAMH 11085		JX460992	KY931895	KY931927
Fluminicola coronata	HKUCC 3717[f]		AF132332	—	—
Gaeumannomyces graminis	AR 3401, M 57		AF362557	JF414874	—
Gelasinospora tetrasperma	CBS 178.33	T	DQ470980	DQ471032	DQ470932
Gnomonia gnomon	CBS 199.53		AF408361	DQ471019	DQ470922
Jattaea algeriensis	CBS 120871[g]	T	EU367457	EU367462	HQ878603
Jobellisia fraterna	S.M.H. 2863		AY346285	—	—
Jobellisia luteola	S.M.H. 2753		AY346286	—	—
Junewangia aquatica	HFJAU0700	T	MG213737	MG213736	—
Lanspora coronata	J.K. 4839A		U46889	DQ470996	DQ470899
Lasiosphaeria ovina	S.M.H. 1538, CBS 958.72		AF064643	AY083799	AY600292
Lentomitella vestita	PRA-12739		KY931820	KY931879	KY931848
Linocarpon elaeidis	HKUCC 5458		DQ810222	DQ810257	—
Macgarvieomyces borealis	CBS 461.65[h]	T	DQ341511	DQ341489	KM485070
Magnaporthe grisea	Ina168, 70-15		AB026819	DQ493955	—
Menispora uncinata	ICMP 18253		GU180637	GU180614	GU180659

Myrmecridium flexuosum	CBS 398.76	T	EU041825	—	—
Myrmecridium schulzeri	CBS 100.54		EU041826	—	—
Natantiella ligneola	CBS 123470		FJ617556	HQ878598	HQ878605
Neoascotaiwania terrestris	CBS 144402		MN699434	MN699386	MN704310
Neolinocarpon globosicarpum	HKUCC 1959		DQ810224	DQ810258	DQ810245
Ophiostoma piliferum	CBS 158.74		DQ470955	DQ471003	DQ470905
Papulosa amerospora	J.K. 5547F		DQ470950	DQ470998	DQ470901
Pararamichloridium livistonae	CBS 144522		MK442542	—	—
Pararamichloridium verrucosum	CBS 128.86	T	EF204508	EF204522	—
Phaeoacremonium fraxinopennsylvanicum	CBS 128920		HQ878595	HQ878600	HQ878609
Phaeoacremonium minimum	CBS 213.31, CBS 111015		AY761082	AY761068	HQ878610
Phomatospora bellaminuta	J.K. 5543N		FJ176857	FJ176803	FJ238345
Pseudodactylaria fusiformis	MFLUCC 20-0085		MT184906	MT184897	MT188555
Pseudodactylaria xanthorrhoeae	CBS 143414	T	MG386117	—	—
Pseudostanjehughesia aquitropica	MFLUCC 16-0569	T	MF077559	MF077537	—
Pseudostanjehughesia lignicola	MFLUCC 15-0352	T	MK849787	—	MN124534
Raffaelea ambrosiae	CBS 185.64	T	EU984297	AY497518	—
Rhamphoria delicatula	CBS 132724		FJ617561	JX066711	JX066702
Rhamphoria pyriformis	CBS 139033		KT991665	MG600406	KT991656
Rhamphoriopsis muriformis	CBS 127683	P	MG600395	MG600403	MG600399
Rhexodenticula acaciae	CBS 142119	T	KY173532	—	—
Rhexodenticula cylindrospora	CBS 318.95	T	KM485039	—	—
Rhodoveronaea varioseptata	CBS 123473		FJ617560	JX066710	JX066700
Riomyces rotundus	AF 303-1	T	JF775589	JF758612	—
Rubellisphaeria abscondita	CBS 132078	T	KT991666	KT991646	KT991657
Sordaria fimicola	S.M.H. 4106, CBS 723.96		AY780079	X69851	DQ368647
Spadicoides bina	CBS 137794		KY931824	KY931881	KY931851
Sporidesmium fluminicola	MFLUCC 15-0346	T	KU376271	—	—
Sporidesmium minigelatinosum	NN47497		DQ408567	—	DQ435090
Sporidesmium submersum	MFLUCC 15-0421	T	KU376272	—	—
Sporoschisma hemipsilum	S.M.H. 2125		AY346292	—	AY780184
Sporothrix schenckii	ATCC MYA-4821		JQ070138	—	—
Tenuimurus clematidis	MFLUCC 14-0833	T	KX549450	—	—
Thyridium oculorum	CBS 110031	T	KJ573449	—	—
Togniniella acerosa	CBS 113648	P	AY761076	AY761073	GU180660
Valsa ambiens	AR 3516		AF362564	DQ862056	DQ862025
Woswasia atropurpurea	CBS 133167	T	JX233658	JX233658	JX233659
Xylochrysis lucida	CBS 135996	T	KF539911	KF539912	KF539913
Xylolentia brunneola	PRA-13611	T	MG600398	MG600407	MG600402
Xylomelasma shoalensis	ILLS 76895	T	KX290919	—	—

Notes:
T, P indicate ex-holotype and ex-paratype strains
[a] paratype of *Cryptadelphia polyseptata*
[b] holotype of *Calosphaeriophora pulchella*
[c] holotype of *Poroconiochaeta discoidea*
[d] holotype of *Coniochaetidium ostreum*
[e] holotype of *Cryptendoxyla hypophloia*
[f] Nom. inval., Art. 36.1(c) (Melbourne)
[g] holotype of *Jattaea prunicola*
[h] holotype of *Diplorhinotrichum juncicola*
