# Supplementary material for: ﻿Re-evaluation of Ceratostomella and Xylomelasma with introduction of two new species (Sordariomycetes)
Source: MycoKeys. 2024 Nov 21;110:319–60. doi: 10.3897/mycokeys.110.136844 (PMC11605300; doi:10.3897/mycokeys.110.136844)
Supplement: Supplementary material 2 — Estimates of evolutionary divergence between ITS rDNA, rpb2 and tef1-α sequences [file mycokeys-110-319-s002.pdf]

Supplementary Table S2. Estimates of evolutionary divergence between ITS rDNA, *rpb2* and *tef1-α* sequences of members of *Ceratostomella*. The numbers of base differences per site between sequences are shown. Analyses were conducted using the Kimura 2-parameter model (Kimura 1980). Evolutionary analyses were conducted in MEGA11 (Tamura et al. 2021).

1. Estimates of evolutionary divergence between ITS rDNA sequences of *Ceratostomella*.
This analysis involved 10 nucleotide sequences. All ambiguous positions were removed for each sequence pair (pairwise deletion option). There were a total of 587 positions in the final dataset.

Species 1	Species 2	Dist
Ceratostomella pyrenaica CBS 129343	Ceratostomella pyrenaica MR3584	0.00000
Ceratostomella pyrenaica CBS 129343	Ceratostomella pyrenaica CBS 117116	0.00000
Ceratostomella pyrenaica MR3584	Ceratostomella pyrenaica CBS 117116	0.00000
Ceratostomella cuspidata ICMP 17629	Ceratostomella cuspidata IFBL 5731	0.00187
Ceratostomella crypta CBS 131683	Ceratostomella crypta CBS 131684	0.00380
Ceratostomella crypta CBS 131683	Ceratostomella sordida CBS 116000	0.07558
Ceratostomella crypta CBS 131684	Ceratostomella sordida CBS 116000	0.07752
Ceratostomella cuspidata ICMP 17629	Ceratostomella pyrenaica CBS 129343	0.19574
Ceratostomella cuspidata IFBL 5731	Ceratostomella pyrenaica CBS 129343	0.19574
Ceratostomella cuspidata ICMP 17629	Ceratostomella pyrenaica MR3584	0.19574
Ceratostomella cuspidata IFBL 5731	Ceratostomella pyrenaica MR3584	0.19574
Ceratostomella cuspidata ICMP 17629	Ceratostomella pyrenaica CBS 117116	0.19574
Ceratostomella cuspidata IFBL 5731	Ceratostomella pyrenaica CBS 117116	0.19574
Ceratostomella cuspidata ICMP 17629	Ceratostomella novaezelandiae PDD 81433	0.19921
Ceratostomella cuspidata IFBL 5731	Ceratostomella novaezelandiae PDD 81433	0.19921
Ceratostomella novaezelandiae PDD 81433	Ceratostomella melanospora CBS 147993	0.20160
Ceratostomella novaezelandiae PDD 81433	Ceratostomella pyrenaica CBS 129343	0.20683
Ceratostomella novaezelandiae PDD 81433	Ceratostomella pyrenaica MR3584	0.20683
Ceratostomella novaezelandiae PDD 81433	Ceratostomella pyrenaica CBS 117116	0.20683
Ceratostomella crypta CBS 131684	Ceratostomella novaezelandiae PDD 81433	0.21042
Ceratostomella novaezelandiae PDD 81433	Ceratostomella sordida CBS 116000	0.21138
Ceratostomella crypta CBS 131683	Ceratostomella novaezelandiae PDD 81433	0.21242
Ceratostomella crypta CBS 131683	Ceratostomella cuspidata ICMP 17629	0.23139
Ceratostomella crypta CBS 131684	Ceratostomella cuspidata ICMP 17629	0.23139
Ceratostomella crypta CBS 131683	Ceratostomella cuspidata IFBL 5731	0.23139
Ceratostomella crypta CBS 131684	Ceratostomella cuspidata IFBL 5731	0.23139
Ceratostomella pyrenaica CBS 129343	Ceratostomella sordida CBS 116000	0.23506
Ceratostomella pyrenaica MR3584	Ceratostomella sordida CBS 116000	0.23506
Ceratostomella pyrenaica CBS 117116	Ceratostomella sordida CBS 116000	0.23506
Ceratostomella crypta CBS 131683	Ceratostomella pyrenaica CBS 129343	0.23669
Ceratostomella crypta CBS 131684	Ceratostomella pyrenaica CBS 129343	0.23669
Ceratostomella crypta CBS 131683	Ceratostomella pyrenaica MR3584	0.23669
Ceratostomella crypta CBS 131684	Ceratostomella pyrenaica MR3584	0.23669
Ceratostomella crypta CBS 131683	Ceratostomella pyrenaica CBS 117116	0.23669
Ceratostomella crypta CBS 131684	Ceratostomella pyrenaica CBS 117116	0.23669
Ceratostomella melanospora CBS 147993	Ceratostomella sordida CBS 116000	0.23695
Ceratostomella crypta CBS 131683	Ceratostomella melanospora CBS 147993	0.23857
Ceratostomella crypta CBS 131684	Ceratostomella melanospora CBS 147993	0.23857
Ceratostomella cuspidata ICMP 17629	Ceratostomella sordida CBS 116000	0.23935
Ceratostomella cuspidata IFBL 5731	Ceratostomella sordida CBS 116000	0.23935
Ceratostomella melanospora CBS 147993	Ceratostomella pyrenaica CBS 129343	0.24152

Ceratostomella melanospora CBS 147993	Ceratostomella pyrenaica MR3584	0.24152
Ceratostomella melanospora CBS 147993	Ceratostomella pyrenaica CBS 117116	0.24152
Ceratostomella cuspidata ICMP 17629	Ceratostomella melanospora CBS 147993	0.24260
Ceratostomella cuspidata IFBL 5731	Ceratostomella melanospora CBS 147993	0.24260

2. Estimates of evolutionary divergence between *rpb2* sequences of members of *Ceratostomella*

This analysis involved 8 nucleotide sequences. All ambiguous positions were removed for each sequence pair (pairwise deletion option). There were a total of 1185 positions in the final dataset.

**Species 1**	**Species 2**	**Dist**
Ceratostomella crypta CBS 131683	Ceratostomella crypta CBS 131684	0.00180
Ceratostomella pyrenaica CBS 129343	Ceratostomella pyrenaica CBS 117116	0.00366
Ceratostomella cuspidata ICMP 17629	Ceratostomella cuspidata IFBL 5731	0.00467
Ceratostomella crypta CBS 131683	Ceratostomella sordida CBS 116000	0.09617
Ceratostomella crypta CBS 131684	Ceratostomella sordida CBS 116000	0.09617
Ceratostomella melanospora CBS 147993	Ceratostomella sordida CBS 116000	0.14973
Ceratostomella crypta CBS 131684	Ceratostomella melanospora CBS 147993	0.15149
Ceratostomella crypta CBS 131683	Ceratostomella melanospora CBS 147993	0.15309
Ceratostomella cuspidata IFBL 5731	Ceratostomella pyrenaica CBS 129343	0.16246
Ceratostomella cuspidata IFBL 5731	Ceratostomella pyrenaica CBS 117116	0.16246
Ceratostomella cuspidata ICMP 17629	Ceratostomella pyrenaica CBS 117116	0.16262
Ceratostomella cuspidata ICMP 17629	Ceratostomella pyrenaica CBS 129343	0.16453
Ceratostomella cuspidata ICMP 17629	Ceratostomella crypta CBS 131684	0.19210
Ceratostomella cuspidata ICMP 17629	Ceratostomella melanospora CBS 147993	0.19338
Ceratostomella cuspidata IFBL 5731	Ceratostomella crypta CBS 131684	0.19379
Ceratostomella cuspidata ICMP 17629	Ceratostomella crypta CBS 131683	0.19430
Ceratostomella cuspidata IFBL 5731	Ceratostomella melanospora CBS 147993	0.19514
Ceratostomella cuspidata IFBL 5731	Ceratostomella crypta CBS 131683	0.19608
Ceratostomella cuspidata ICMP 17629	Ceratostomella sordida CBS 116000	0.19891
Ceratostomella melanospora CBS 147993	Ceratostomella pyrenaica CBS 117116	0.20126
Ceratostomella cuspidata IFBL 5731	Ceratostomella sordida CBS 116000	0.20219
Ceratostomella melanospora CBS 147993	Ceratostomella pyrenaica CBS 129343	0.20475
Ceratostomella crypta CBS 131684	Ceratostomella pyrenaica CBS 117116	0.20724
Ceratostomella crypta CBS 131683	Ceratostomella pyrenaica CBS 117116	0.20934
Ceratostomella crypta CBS 131684	Ceratostomella pyrenaica CBS 129343	0.21087
Ceratostomella crypta CBS 131683	Ceratostomella pyrenaica CBS 129343	0.21298
Ceratostomella pyrenaica CBS 129343	Ceratostomella sordida CBS 116000	0.21421
Ceratostomella pyrenaica CBS 117116	Ceratostomella sordida CBS 116000	0.21530

3. Estimates of evolutionary divergence between *tef1-α* sequences of *Ceratostomella*.

This analysis involved 8 nucleotide sequences. All ambiguous positions were removed for each sequence pair (pairwise deletion option). There were a total of 996 positions in the final dataset

Species 1	Species 2	Dist
Ceratostomella crypta CBS 131684	Ceratostomella crypta CBS 131683	0.00000
Ceratostomella cuspidata ICMP 17629	Ceratostomella cuspidata IFBL 5731	0.00000
Ceratostomella pyrenaica MR3584	Ceratostomella pyrenaica CBS 117116	0.00000
Ceratostomella crypta CBS 131684	Ceratostomella sordida CBS 116000	0.03651
Ceratostomella crypta CBS 131683	Ceratostomella sordida CBS 116000	0.03746
Ceratostomella crypta CBS 131684	Ceratostomella melanospora CBS 147993	0.06151
Ceratostomella crypta CBS 131683	Ceratostomella melanospora CBS 147993	0.06190
Ceratostomella cuspidata ICMP 17629	Ceratostomella pyrenaica MR3584	0.06435
Ceratostomella cuspidata IFBL 5731	Ceratostomella pyrenaica MR3584	0.06435
Ceratostomella melanospora CBS 147993	Ceratostomella sordida CBS 116000	0.06469
Ceratostomella cuspidata IFBL 5731	Ceratostomella pyrenaica CBS 117116	0.06497
Ceratostomella cuspidata ICMP 17629	Ceratostomella pyrenaica CBS 117116	0.06504
Ceratostomella crypta CBS 131684	Ceratostomella cuspidata IFBL 5731	0.07295
Ceratostomella crypta CBS 131684	Ceratostomella cuspidata ICMP 17629	0.07302
Ceratostomella crypta CBS 131683	Ceratostomella cuspidata ICMP 17629	0.07492
Ceratostomella crypta CBS 131683	Ceratostomella cuspidata IFBL 5731	0.07492
Ceratostomella cuspidata ICMP 17629	Ceratostomella melanospora CBS 147993	0.07635
Ceratostomella cuspidata IFBL 5731	Ceratostomella melanospora CBS 147993	0.07635
Ceratostomella crypta CBS 131683	Ceratostomella pyrenaica MR3584	0.07700
Ceratostomella crypta CBS 131684	Ceratostomella pyrenaica MR3584	0.07700
Ceratostomella crypta CBS 131684	Ceratostomella pyrenaica CBS 117116	0.07817
Ceratostomella crypta CBS 131683	Ceratostomella pyrenaica CBS 117116	0.07908
Ceratostomella melanospora CBS 147993	Ceratostomella pyrenaica MR3584	0.07914
Ceratostomella melanospora CBS 147993	Ceratostomella pyrenaica CBS 117116	0.07953
Ceratostomella pyrenaica MR3584	Ceratostomella sordida CBS 116000	0.08017
Ceratostomella pyrenaica CBS 117116	Ceratostomella sordida CBS 116000	0.08094
Ceratostomella cuspidata ICMP 17629	Ceratostomella sordida CBS 116000	0.08180
Ceratostomella cuspidata IFBL 5731	Ceratostomella sordida CBS 116000	0.08180
