# Supplementary material for: ﻿Re-evaluation of Ceratostomella and Xylomelasma with introduction of two new species (Sordariomycetes)
Source: MycoKeys. 2024 Nov 21;110:319–60. doi: 10.3897/mycokeys.110.136844 (PMC11605300; doi:10.3897/mycokeys.110.136844)
Supplement: Supplementary material 3 — Biogeographical distribution, substrate, habitat and other detailed metadata for Ceratostomella species with available ITS sequences inferred from the GlobalFungi database [file mycokeys-110-319-s003.pdf]

Supplementary Table S3. Biogeographical distribution, substrate, habitat and other detailed metadata for *Ceratostomella* species with available ITS sequences inferred from the GlobalFungi database.

Ceratostomella crypta

id	paper	permanent id	sample type	latitude	longitude	continent	year of sampling from	year of sampling to	Biome	MAT	MAP	pH	SOC	ITS total	abundances
2223	261	GF05006572S	soil	35.3832	-78.0389	North America	2016	2016	cropland	16.7	1192	05.4	07.4	390254	4
14216	324	GF05000593S	soil	30.2622	-89.9568	North America	2017	2017	wetland	20.4	1556	05.5	22.3	112540	15
26963	324	GF05000570S	soil	30.0619	-89.8652	North America	2017	2017	wetland	20.7	1577	05.6	15.2	24425	5
28961	571	GF03003581S	water	34.7181	-76.6707	North America	2012	2012	aquatic	18.8	1397	NA	NA	133373	10
30816	643	GF02000202S	soil	35.84	-83.96	North America	2014	2014	forest	14.9	1192	05.7	05.7	47232	2
35404	164	GF05015945S	soil	28.11	-81.71	North America	2020	2020	cropland	21.5	1304	05.3	17.9	18492	4
36547	99	GF05022033S	air	45.08	-73.38	North America	2010	2010	cropland	07.7	907	06.1	16.9	107002	5
36822	571	GF03003607S	water	34.7181	-76.6707	North America	2013	2013	aquatic	18.8	1397	NA	NA	24024	8
42508	236	GF05010162S	soil	35.6921	-78.4679	North America	2018	2018	anthropogenic	16.2	1093	05.2	07.5	63379	1
46983	138	GF05018154S	sediment	34.176	-118.474	North America	2017	2017	shrubland	18.6	362	07.3	01.5	16360	2

Ceratostomella cuspidata

id	paper	permanent id	sample type	latitude	longitude	continent	year of sampling from	year of sampling to	Biome	MAT	MAP	pH	SOC	ITS total	abundances
54	835	GF01000743S	soil	-43.1094	146.837	Australia	2015	2015	forest	11.2	1106	5	9.7	151915	1
303	835	GF01000742S	soil	-43.0918	146.644	Australia	2015	2015	forest	10.9	1167	5	18.7	121157	1
643	313	GF05001729S	air	-36.9162	174.646	Australia	2017	2017	anthropogenic	15.3	1126	5.4	7.1	424873	5
1333	835	GF01000855S	soil	-28.2887	152.425	Australia	2014	2014	forest	15.8	1044	5.7	2.9	606745	6
2308	313	GF05001728S	air	-36.9162	174.646	Australia	2017	2017	anthropogenic	15.3	1126	5.4	7.1	152660	1
2393	801	GF01005998S	soil	-43.0726	146.809	Australia	2012	2012	forest	11.9	846	4.9	10.7	307319	1
2972	835	GF01001660S	soil	-28.2887	152.425	Australia	2014	2014	forest	15.8	1044	5.7	2.9	342534	20
3414	313	GF05001725S	air	-36.9162	174.646	Australia	2017	2017	anthropogenic	15.3	1126	5.4	7.1	80282	1
3505	835	GF01000870S	soil	-43.0918	146.644	Australia	2015	2015	forest	10.9	1167	5	18.7	108681	3
4156	835	GF01001122S	soil	-43.0951	146.654	Australia	2013	2013	forest	11.7	1026	4.9	17	391265	1
4171	835	GF01001125S	soil	-30.8849	132.197	Australia	2016	2016	shrubland	18.2	229	7.5	0	77617	1
5086	835	GF01001228S	soil	-43.0951	146.654	Australia	2013	2013	forest	11.7	1026	4.9	17	210466	3
7053	835	GF01001324S	soil	-43.0951	146.654	Australia	2013	2013	forest	11.7	1026	4.9	17	164539	13
7096	835	GF01001670S	soil	-43.0951	146.654	Australia	2013	2013	forest	11.7	1026	4.9	17	152926	3
9777	190	GF05013648S	soil	-37.6514	145.83	Australia	2017	2017	forest	12.2	1086	5.1	13.3	80224	5
12943	801	GF01006012S	soil	-33.6478	150.275	Australia	2012	2012	forest	11.5	1242	5.3	2.1	261280	2
13449	190	GF05013656S	soil	-37.6507	145.821	Australia	2017	2017	forest	11.3	1149	5.2	11.7	76222	1
13457	190	GF05013649S	soil	-37.6493	145.821	Australia	2017	2017	forest	10.5	1204	5.3	11.2	60503	2
13511	835	GF01001772S	soil	-43.0455	146.741	Australia	2015	2015	forest	11.8	817	5.2	10.9	137522	1
16062	835	GF01001331S	soil	-41.6556	145.082	Australia	2015	2015	forest	12.5	1457	4.7	8.9	141586	1
22344	313	GF05001724S	air	-36.9162	174.646	Australia	2017	2017	anthropogenic	15.3	1126	5.4	7.1	221407	2
23054	313	GF05001742S	air	-36.9162	174.646	Australia	2017	2017	anthropogenic	15.3	1126	5.4	7.1	417565	1
29047	694	GF01014542S	soil	-43.093	146.65	Australia	2012	2012	forest	11.7	1026	4.9	13.5	5241	4
36644	694	GF01014583S	soil	-37.4883	145.833	Australia	2011	2011	forest	10.1	1305	5.2	10.4	7153	1
43424	835	GF01002505S	soil	-34.8581	148.567	Australia	2013	2013	grassland	13.8	812	6.1	2.9	26279	3
44640	311	GF05001913S	soil	-2.08534	102.79	Asia	2016	2016	cropland	26.1	2977	4.9	13	35874	2
49455	694	GF01014653S	soil	-43.7624	169.389	Australia	2012	2012	forest	11.3	3654	4.8	18.2	5675	4
49888	194	GF05013238S	soil	-43.0928	146.65	Australia	2014	2014	forest	11.7	1026	4.9	13.5	13292	2

Ceratostomella melanospora

id	paper	permanent id	sample type	latitude	longitude	continent	year of sampling from	year of sampling to	Biome	MAT	MAP	pH	SOC	ITS total	abundances
11514	314	GF05001601S	air	56.0738	13.2333	Europe	2007	2007	anthropogenic	7.7	679	5.9	13	495495	1
13123	210	GF05012347S	soil	42.1667	128.1	Asia	2017	2017	forest	1.9	963	5.6	16.1	38288	1
15326	210	GF05012442S	soil	42.1667	128.1	Asia	2017	2017	forest	1.9	963	5.6	16.1	34856	2
24724	181	GF05014480S	air	45.2487	8.69997	Europe	2016	2016	cropland	14	869	7	6.7	47795	2
25990	99	GF05021990S	air	49.24	-121.76	North America	2011	2011	cropland	11.4	1578	5.7	9.8	99297	1
47700	210	GF05012368S	soil	42.1667	128.1	Asia	2017	2017	forest	1.9	963	5.6	16.1	30308	1
53998	181	GF05014478S	air	45.2487	8.69997	Europe	2016	2016	cropland	14	869	7	6.7	13068	3

Ceratostomella novae-zelandiae

id	paper	permanent id	sample type	latitude	longitude	continent	year of sampling from	year of sampling to	Biome	MAT	MAP	pH	SOC	ITS total	abundances
956	11	GF05030149S	soil	-40.7767	-72.1978	South America	2014	2014	forest	5.9	2388	5.3	16.6	414434	4
8085	801	GF01006006S	soil	-37.9469	175.077	Australia	2014	2014	forest	13.4	1563	5	3.7	66750	1

Ceratostomella pyrenaica

id	paper	permanent id	sample type	latitude	longitude	continent	year of sampling from	year of sampling to	Biome	MAT	MAP	pH	SOC	ITS total	abundances
762	84	GF05022654S	deadwood	45.1426	7.10272	Europe	2015	2015	forest	10.2	585	6.5	7	499511	1
2183	564	GF03004890S	soil	47.9218	20.191	Europe	2017	2017	forest	10.2	541	6.7	4.2	65023	1
3842	140	GF05017969S	shoot	21.3095	-157.762	North America	2011	2011	woodland	22.2	980	5.2	9.8	1828	1
4733	371	GF04017762S	soil	35.049	-83.434	North America	2013	2013	forest	11.9	1550	5.1	20.6	115580	43
5883	175	GF05015043S	root	35.4883	-80.0732	North America	2012	2012	forest	15.8	1188	4.9	3.4	36790	1
6161	643	GF02000048S	rhizosphere soil	35.84	-83.96	North America	2014	2014	forest	14.9	1192	5.7	5.7	212418	1
6460	275	GF05005266S	soil	47.18	7.4	Europe	2017	2017	cropland	10.5	1083	6.5	9.3	48612	2
7491	298	GF05002719S	shoot	42.4108	-85.3729	North America	2018	2018	cropland	9.2	882	6.1	10.6	105000	3
7630	175	GF05015060S	root	41.9601	-75.717	North America	2012	2012	forest	9.3	902	5	25	37148	1
7949	722	GF01012119S	soil	36.7701	12.0247	Europe	2013	2013	forest	16.4	512	6.6	5.2	302510	1
10441	590	GF03000962S	soil	36.5614	52.0445	Asia	2018	2018	forest	19.2	891	6.6	4.4	134027	1
10767	643	GF02000148S	shoot	35.84	-83.96	North America	2014	2014	forest	14.9	1192	5.7	5.7	187269	1
10776	643	GF02000075S	shoot	35.84	-83.96	North America	2014	2014	forest	14.9	1192	5.7	5.7	172455	1
11383	710	GF01012857S	shoot	46.7947	8.42	Europe	2013	2013	forest	10.1	984	5.6	15.9	51797	2
13121	312	GF04015664S	soil	49.6684	14.5822	Europe	2017	2017	forest	8.4	524	5.7	13.7	14246	1
14524	371	GF04017776S	soil	35.051	-83.4342	North America	2013	2013	forest	12.4	1494	5	16.1	121948	1
15529	84	GF05022660S	deadwood	45.1722	7.73	Europe	2015	2015	forest	12.6	773	6.2	6.7	64771	57
16253	703	GF01013495S	soil	46.5878	11.47	Europe	2015	2015	forest	3	987	5.3	21.6	138555	1
17008	236	GF05010198S	soil	35.8759	-78.6179	North America	2018	2018	anthropogenic	15.6	1222	5.3	7.4	86445	5
18932	181	GF05014497S	air	45.2487	8.69997	Europe	2016	2016	cropland	14	869	7	6.7	24653	1
19187	583	GF03002028S	soil	35.6942	-83.5065	North America	2017	2017	forest	13.1	1215	5.2	10.2	51248	1
20320	710	GF01012908S	shoot	45.9645	8.76	Europe	2013	2013	forest	11.6	1466	6.1	3.9	111329	2
21212	175	GF05014888S	soil	41.4384	-90.5554	North America	2012	2012	forest	10.3	848	6.1	5.5	49871	2
22061	181	GF05014513S	air	45.2487	8.69997	Europe	2016	2016	cropland	14	869	7	6.7	34822	1
23481	710	GF01012935S	shoot	47.4734	8.337	Europe	2013	2013	forest	10	1238	5.9	14.8	43233	5
23587	181	GF05014475S	air	45.2487	8.69997	Europe	2016	2016	cropland	14	869	7	6.7	59009	2
24061	63	GF05024518S	shoot	45.8444	7.57806	Europe	2018	2018	grassland	1.2	783	5.7	14.6	146011	31
24151	371	GF04017787S	soil	35.5879	-83.0808	North America	2013	2013	forest	8.3	1552	4.7	25.7	131317	1
24724	181	GF05014480S	air	45.2487	8.69997	Europe	2016	2016	cropland	14	869	7	6.7	47795	1
28109	564	GF03004901S	soil	47.9163	20.1843	Europe	2017	2017	forest	9.9	534	6.9	4.7	46407	1
29507	181	GF05014512S	air	45.2487	8.69997	Europe	2016	2016	cropland	14	869	7	6.7	51998	2
29664	371	GF04017795S	soil	35.049	-83.434	North America	2013	2013	forest	11.9	1550	5.1	20.6	99341	1
30150	371	GF04017796S	soil	35.0382	-83.4594	North America	2013	2013	forest	10.2	1873	4.9	24.6	96848	1
31467	371	GF04017773S	soil	35.0441	-83.4572	North America	2013	2013	forest	11.5	1603	5	21.1	107967	1
32578	181	GF05014479S	air	45.2487	8.69997	Europe	2016	2016	cropland	14	869	7	6.7	39569	4
33231	50	GF05025911S	soil	45.85	9	Europe	2016	2016	cropland	12.1	1192	6.6	5.8	29334	1
33471	181	GF05014386S	air	45.2487	8.69997	Europe	2016	2016	cropland	14	869	7	6.7	21961	2
36889	262	GF05006484S	soil	38.6219	-8.13936	Europe	2018	2018	grassland	15.7	710	6.3	6.3	29405	1
38690	181	GF05014506S	air	45.2487	8.69997	Europe	2016	2016	cropland	14	869	7	6.7	39854	1
40756	739	GF01010027S	soil	49.2559	16.6451	Europe	2013	2013	forest	9	544	5	12.4	9887	1
45493	80	GF05023126S	soil	35.895	-77.679	North America	2018	2018	grassland	16.2	1168	5	17	42787	3
46366	181	GF05014553S	air	45.2487	8.69997	Europe	2016	2016	cropland	14	869	7	6.7	29626	2
46925	181	GF05014505S	air	45.2487	8.69997	Europe	2016	2016	cropland	14	869	7	6.7	29507	1
47138	225	GF05011118S	soil	35	-83.5	North America	2012	2012	forest	9.2	2371	4.6	30.8	36730	1
47572	181	GF05014594S	air	45.2487	8.69997	Europe	2016	2016	cropland	14	869	7	6.7	47069	1

48822	564	GF03004899S	soil	47.9127	20.1819	Europe	2017	2017	forest	10.3	537	6.7	5.4	56794	1
50191	1	GF05032070S	soil	45.7616	16.6982	Europe	2018	2018	woodland	11.6	890	5.9	6.8	6720	1
51009	181	GF05014554S	air	45.2487	8.69997	Europe	2016	2016	cropland	14	869	7	6.7	31215	3
53998	181	GF05014478S	air	45.2487	8.69997	Europe	2016	2016	cropland	14	869	7	6.7	13068	1
54025	181	GF05014507S	air	45.2487	8.69997	Europe	2016	2016	cropland	14	869	7	6.7	29719	3

Ceratostomella sordida

id	paper	permanent id	sample type	latitude	longitude	continent	year of sampling from	year of sampling to	Biome	MAT	MAP	pH	SOC	ITS total	abundances
962	314	GF05001630S	air	67.8558	20.2253	Europe	2007	2007	forest	-1.1	574	5.4	16.2	629870	1
1082	498	GF03009220S	dust	37.4653	126.955	Asia	2015	2015	anthropogenic	12	1383	5.5	6	94732	4
1306	118	GF05019702S	root	38.43	109.02	Asia	2018	2018	desert	9	354	8.4	1.1	45392	1
2772	181	GF05014457S	air	45.2487	8.69997	Europe	2016	2016	cropland	14	869	7	6.7	46556	1
3925	566	GF03004612S	soil	46.4056	10.45	Europe	2015	2015	tundra	-1.5	960	NA	NA	583538	15
4287	314	GF05001646S	air	56.0738	13.2333	Europe	2007	2007	anthropogenic	7.7	679	5.9	13	417224	37
4890	311	GF05001971S	soil	-1.90214	103.381	Asia	2016	2016	cropland	26.3	2335	4.4	8.1	44981	1
5809	635	GF02001033S	soil	36.05	108.31	Asia	2015	2015	grassland	9.1	505	7.6	10.9	51040	7
6865	314	GF05001684S	air	67.8558	20.2253	Europe	2006	2006	forest	-1.1	574	5.4	16.2	277266	1
7043	391	GF04007257S	water	34.1649	-117.682	North America	2018	2018	aquatic	16.9	538	6.9	2.9	68042	1
7163	498	GF03009237S	dust	37.4653	126.955	Asia	2015	2015	anthropogenic	12	1383	5.5	6	119920	1
7334	99	GF05022031S	air	46.34	-63.17	North America	2010	2010	cropland	5.7	1196	4.9	16.3	91232	1
7414	665	GF01018233S	soil	10.04	-85.6129	North America	2013	2013	forest	25.2	1716	5.9	7.1	84852	2
7505	314	GF05001676S	air	67.8558	20.2253	Europe	2006	2006	forest	-1.1	574	5.4	16.2	271039	41
7770	498	GF03009238S	dust	37.4653	126.955	Asia	2015	2015	anthropogenic	12	1383	5.5	6	105736	6
7773	236	GF05010192S	soil	36.1206	-78.8577	North America	2018	2018	anthropogenic	15.7	1059	5.5	5.2	213611	7
7914	498	GF03009263S	air	37.4653	126.955	Asia	2015	2015	anthropogenic	12	1383	5.5	6	64178	4
9147	665	GF01018242S	soil	10.865	-85.5742	North America	2013	2013	forest	25.7	1739	5.7	6.7	83800	1
9357	566	GF03004620S	soil	46.4064	10.584	Europe	2015	2015	tundra	-1.5	960	NA	NA	440343	1
9438	641	GF02000435S	soil	44.8	-96.57	North America	2003	2015	grassland	6.4	595	6.8	3.6	92171	4
9651	221	GF05011258S	soil	43.1034	-85.7286	North America	2017	2017	cropland	8.7	841	6.1	10.4	115910	38
10775	643	GF02000071S	shoot	35.84	-83.96	North America	2014	2014	forest	14.9	1192	5.7	5.7	186934	1
11204	312	GF05001252V	soil	26.94	-81.34	North America	2013	2013	forest	NA	NA	6.1	17.5	23292	2
11298	236	GF05010229S	soil	35.9462	-78.945	North America	2018	2018	anthropogenic	15.9	1137	5.1	7.1	200087	1
11514	314	GF05001601S	air	56.0738	13.2333	Europe	2007	2007	anthropogenic	7.7	679	5.9	13	495495	2
11900	736	GF01010272S	deadwood	29.2425	118.1	Asia	2012	2012	forest	16.8	1748	5.4	9.1	3587	84
12102	324	GF05000607S	soil	30.3341	-90.0333	North America	2017	2017	wetland	20.2	1596	5.2	9.9	70878	15
12763	635	GF02001039S	soil	36.05	108.31	Asia	2015	2015	grassland	9.1	505	7.6	10.9	50085	1
12940	156	GF05016520S	shoot	43.3388	-4.32634	Europe	2017	2017	anthropogenic	14.7	1145	6	6.8	47542	3
13951	156	GF05016546S	shoot	46.5115	6.63	Europe	2017	2017	anthropogenic	9.7	1196	5.9	21.1	63647	1
14638	312	GF05000146V	soil	-6.36	145.28	Australia	2011	2011	forest	NA	NA	5.1	9.8	13221	1
14783	312	GF05001358V	soil	4.94	-52.3	South America	2013	2013	forest	NA	NA	5.4	12.4	10842	1
14793	312	GF05001336V	soil	36.3	52.9	Asia	2012	2012	forest	NA	NA	6.7	9.9	13710	1
14840	51	GF05025217S	soil	0	35.535	Africa	2017	2018	shrubland	14	1834	5.1	9.5	52394	1
15711	391	GF04007260S	water	29.6975	-81.9817	North America	2018	2018	aquatic	20.6	1268	NA	NA	99576	1
15759	181	GF05014527S	air	45.2487	8.69997	Europe	2016	2016	cropland	14	869	7	6.7	61229	12
16441	665	GF01018278S	soil	10.93	-85.5973	North America	2013	2013	forest	25.2	2036	5.7	7.4	81458	76
16701	710	GF01012873S	shoot	47.4734	8.337	Europe	2013	2013	forest	10	1238	5.9	14.8	111875	7
17013	51	GF05025175S	soil	-27.8805	29.9649	Africa	2017	2018	grassland	16.9	694	6.2	0	35348	1
17221	221	GF05011264S	soil	43.1256	-85.364	North America	2017	2017	cropland	8.7	807	6.2	12.9	98081	1
18215	498	GF03009256S	dust	37.4653	126.955	Asia	2015	2015	anthropogenic	12	1383	5.5	6	154671	1
18977	181	GF05014526S	air	45.2487	8.69997	Europe	2016	2016	cropland	14	869	7	6.7	32089	8
19924	439	GF04003276S	soil	34.87	114.33	Asia	2017	2017	cropland	15	619	7.6	7.5	47271	4
20511	525	GF03007590S	soil	47.29	26.5	Europe	2017	2017	forest	6.3	704	5.7	14.6	54534	1
20728	164	GF05015911S	soil	28.11	-81.71	North America	2019	2019	cropland	21.5	1304	5.3	17.9	18954	1

20729	261	GF05006566S	soil	35.3832	-78.0389	North America	2016	2016	cropland	16.7	1192	5.4	7.4	395844	2
22344	313	GF05001724S	air	-36.9162	174.646	Australia	2017	2017	anthropogenic	15.3	1126	5.4	7.1	221407	3
22547	439	GF04003310S	soil	29.8	118.26	Asia	2017	2017	cropland	17.5	1912	5.9	17.1	78384	1
22945	779	GF01007626S	soil	-30.5611	29.8144	Africa	2015	2015	grassland	16	891	5.5	5.1	32850	1
23821	99	GF05021981S	air	49.24	-121.76	North America	2011	2011	cropland	11.4	1578	5.7	9.8	65124	1
24525	710	GF01012952S	shoot	45.8731	10.03	Europe	2013	2013	forest	9.2	1005	5.4	12.9	117898	6
24645	498	GF03009264S	dust	37.4653	126.955	Asia	2015	2015	anthropogenic	12	1383	5.5	6	89511	1
24724	181	GF05014480S	air	45.2487	8.69997	Europe	2016	2016	cropland	14	869	7	6.7	47795	1
25469	164	GF05015920S	soil	28.11	-81.71	North America	2019	2019	cropland	21.5	1304	5.3	17.9	13284	4
25994	182	GF05014376S	shoot	-33.9354	18.7516	Africa	2019	2019	cropland	16.5	1073	6.4	10.8	127589	2
26106	99	GF05021973S	air	45.08	-73.38	North America	2011	2011	cropland	7.7	907	6.1	16.9	94059	1
26398	439	GF04003340S	soil	37.55	118.77	Asia	2017	2017	cropland	13.1	564	7.9	6.2	65581	1
26413	573	GF03003464S	rhizosphere soil	42.5406	-2.77389	Europe	2017	2017	cropland	13	626	7.8	2.8	102358	4
27870	210	GF05012450S	soil	42.1667	128.1	Asia	2017	2017	forest	1.9	963	5.6	16.1	39475	1
28894	181	GF05014485S	air	45.2487	8.69997	Europe	2016	2016	cropland	14	869	7	6.7	31558	5
29347	236	GF05010226S	soil	35.9462	-78.945	North America	2018	2018	anthropogenic	15.9	1137	5.1	7.1	107458	1
29357	261	GF05006573S	soil	35.3832	-78.0389	North America	2016	2016	cropland	16.7	1192	5.4	7.4	287755	2
29551	665	GF01018279S	soil	10.93	-85.5973	North America	2013	2013	forest	25.2	2036	5.7	7.4	75851	24
29960	439	GF04003394S	soil	24.95	118.7	Asia	2017	2017	cropland	21.7	1009	6.4	12.6	43147	1
30765	390	GF04007419S	litter	-1.78038	-51.4285	South America	2015	2015	forest	26.5	2118	NA	NA	9146	1
30901	837	GF01000695S	root	-25.8669	-54.1797	South America	2013	2013	cropland	20.9	1982	5.4	3.2	31530	1
31253	164	GF05015918S	soil	28.11	-81.71	North America	2019	2019	cropland	21.5	1304	5.3	17.9	25946	3
31623	178	GF05014690S	soil	43.7047	-92.5644	North America	2014	2014	grassland	7.4	793	6	4.3	22746	1
32111	665	GF01018295S	soil	10.15	-85.5755	North America	2013	2013	forest	25.5	1921	5.9	6.8	156050	31
32603	78	GF05023497S	soil	28.129	-81.716	North America	2018	2018	cropland	21.5	1288	5.2	16.9	34479	1
33405	180	GF05014603S	air	40.4081	116.674	Asia	2018	2018	anthropogenic	11.9	563	7.5	7.6	94240	4
33849	99	GF05022018S	air	45.08	-73.38	North America	2011	2011	cropland	7.7	907	6.1	16.9	71334	1
34365	194	GF05013090S	soil	-25.1008	-47.9275	South America	2019	2019	forest	21.5	2812	4.9	1.2	9665	3
35244	571	GF03003596S	water	34.7181	-76.6707	North America	2011	2011	aquatic	18.8	1397	NA	NA	104167	7
35558	351	GF04019458S	soil	39.969	115.432	Asia	2015	2015	forest	5.8	559	6.5	15.5	63108	2
36226	164	GF05015930S	soil	28.11	-81.71	North America	2020	2020	cropland	21.5	1304	5.3	17.9	19622	5
36268	387	GF04007927S	root	34.9779	-97.5228	North America	2015	2015	grassland	16.7	929	6	18.6	26341	1
36547	99	GF05022033S	air	45.08	-73.38	North America	2010	2010	cropland	7.7	907	6.1	16.9	107002	2
36669	164	GF05015961S	soil	28.11	-81.71	North America	2019	2019	cropland	21.5	1304	5.3	17.9	23214	3
36890	643	GF02000111S	rhizosphere soil	35.84	-83.96	North America	2014	2014	forest	14.9	1192	5.7	5.7	125231	1
37285	180	GF05014607S	air	40.4081	116.674	Asia	2019	2019	anthropogenic	11.9	563	7.5	7.6	85841	1
38643	665	GF01018311S	soil	10.04	-85.6129	North America	2013	2013	forest	25.2	1716	5.9	7.1	51396	2
38812	181	GF05014448S	air	45.2487	8.69997	Europe	2016	2016	cropland	14	869	7	6.7	31850	1
38903	312	GF05001356V	soil	26.4	-80.11	North America	2013	2013	forest	NA	NA	NA	NA	21555	1
39709	81	GF05023061S	soil	31.14	121.29	Asia	2017	2017	anthropogenic	16.3	1154	7.1	16.8	64451	1
40108	210	GF05012335S	soil	42.1667	128.1	Asia	2017	2017	forest	1.9	963	5.6	16.1	40448	1
40207	351	GF04019499S	soil	31.6225	110.32	Asia	2015	2015	forest	6.7	1227	5.6	24.8	55899	2
40537	736	GF01010296S	deadwood	29.2496	118.12	Asia	2012	2012	forest	16.4	1949	5	16.5	5609	2
42201	236	GF05010230S	soil	35.7045	-80.5003	North America	2018	2018	anthropogenic	15.5	1077	5.4	6.6	111111	1
42363	164	GF05015971S	soil	28.11	-81.71	North America	2019	2019	cropland	21.5	1304	5.3	17.9	18832	1
43366	686	GF01016030S	rhizosphere soil	40.5057	-77.9976	North America	2012	2012	forest	11.2	894	5.4	16.9	5354	2
44601	210	GF05012404S	soil	42.1667	128.1	Asia	2017	2017	forest	1.9	963	5.6	16.1	32715	2
46748	74	GF05023589S	rhizosphere soil	41.54	125.61	Asia	2017	2017	cropland	6.6	794	5.6	6.2	42563	2
48458	312	GF05001523V	soil	55.91	92.75	Europe	2016	2016	forest	NA	NA	4.9	28.1	12296	1

49153	164	GF05015960S	soil	28.11	-81.71	North America	2019	2019	cropland	21.5	1304	5.3	17.9	23898	1
50344	351	GF04019568S	soil	30.3489	119.426	Asia	2015	2015	forest	10.9	2237	5.6	26.4	73501	1
51045	837	GF01000700S	root	-25.8544	-53.9309	South America	2013	2013	cropland	20.8	1990	5.4	3	28148	3
51251	174	GF05015142S	soil	37.2436	-6.26357	Europe	2018	2018	grassland	18.9	558	6.7	3.9	76792	3
52020	181	GF05014403S	air	45.2487	8.69997	Europe	2016	2016	cropland	14	869	7	6.7	38640	2
52832	312	GF05000780V	soil	29.74	-82.44	North America	2012	2012	forest	NA	NA	5.1	9.3	10392	1
53935	164	GF05015933S	soil	28.11	-81.71	North America	2020	2020	cropland	21.5	1304	5.3	17.9	18236	2
54163	815	GF01005102S	deadwood	35.04	135.45	Asia	2009	2009	cropland	13.4	1442.4	4.9	17.7	2004	7
54164	815	GF01005061S	deadwood	35.51	137.55	Asia	2013	2013	forest	13	1524	5.2	10.7	2434	40
55992	91	GF05022565S	soil	42.5385	-76.4973	North America	2014	2014	grassland	9.2	1039	6.3	17.8	8591	2
56101	164	GF05015913S	soil	28.11	-81.71	North America	2019	2019	cropland	21.5	1304	5.3	17.9	13251	15
56378	351	GF04019367S	soil	42.3777	128.352	Asia	2016	2016	forest	4	740	5.4	14.9	34297	1
56497	164	GF05015928S	soil	28.11	-81.71	North America	2020	2020	cropland	21.5	1304	5.3	17.9	16870	9
56926	99	GF05022046S	air	46.34	-63.17	North America	2010	2010	cropland	5.7	1196	4.9	16.3	21704	1
57323	259	GF05006944S	shoot	43.1167	-0.533333	Europe	2016	2016	forest	12.6	1377	5.1	11	18961	1
57503	736	GF01010325S	deadwood	29.2145	118.137	Asia	2012	2012	forest	16.5	2064	5.2	16.7	3960	38
57565	725	GF01012010S	soil	48.23	7.25	Europe	2010	2010	forest	7	1077	4.3	22.6	3425	1
57607	736	GF01010326S	deadwood	29.2814	118.088	Asia	2012	2012	forest	15.7	2015	5.3	8.5	5226	63
57828	164	GF05015949S	soil	28.11	-81.71	North America	2020	2020	cropland	21.5	1304	5.3	17.9	25676	2
58103	324	GF05000538S	soil	29.7786	-90.1307	North America	2017	2017	wetland	21	1564	5.9	20	14209	3
59984	736	GF01010335S	deadwood	29.2814	118.088	Asia	2012	2012	forest	15.7	2015	5.3	8.5	5026	74
60337	815	GF01005058S	deadwood	38.14	140.21	Asia	2012	2012	forest	10.8	1329	5	31.4	2718	2
62082	582	GF03002117S	shoot	27.58	99	Asia	2017	2017	cropland	11	760	5.9	21.5	3376	1
62316	111	GF05020258S	soil	47.4833	14.1	Europe	2016	2016	grassland	7.8	860	5.4	21.8	3001	2
64051	736	GF01010355S	deadwood	29.255	118.147	Asia	2012	2012	forest	14.3	2568	5	11.6	4152	3
66269	736	GF01010380S	deadwood	29.2493	118.135	Asia	2012	2012	forest	14.8	2407	5.1	15.9	4574	80
66359	736	GF01010366S	deadwood	29.2145	118.137	Asia	2012	2012	forest	16.5	2064	5.2	16.7	4715	7
67418	736	GF01010377S	deadwood	29.2496	118.12	Asia	2012	2012	forest	16.4	1949	5	16.5	4293	7
71972	736	GF01010360S	deadwood	29.2425	118.1	Asia	2012	2012	forest	16.8	1748	5.4	9.1	4742	196
72675	815	GF01005097S	deadwood	38.14	140.21	Asia	2012	2012	forest	10.8	1329	5	31.4	2934	12
73471	815	GF01005103S	deadwood	34.47	135.5	Asia	2012	2012	forest	16.5	1448	5.9	18.1	2490	16
75641	312	GF05000867V	soil	37.74	126.53	Asia	2019	2019	forest	NA	NA	5.7	8.1	5298	2
